# An integrated gene catalog and over 10,000 metagenome-assembled genomes from the gastrointestinal microbiome of ruminants

**DOI:** 10.1186/s40168-021-01078-x

**Published:** 2021-06-12

**Authors:** Fei Xie, Wei Jin, Huazhe Si, Yuan Yuan, Ye Tao, Junhua Liu, Xiaoxu Wang, Chengjian Yang, Qiushuang Li, Xiaoting Yan, Limei Lin, Qian Jiang, Lei Zhang, Changzheng Guo, Chris Greening, Rasmus Heller, Le Luo Guan, Phillip B. Pope, Zhiliang Tan, Weiyun Zhu, Min Wang, Qiang Qiu, Zhipeng Li, Shengyong Mao

**Affiliations:** 1 Laboratory of Gastrointestinal Microbiology, College of Animal Science and Technology, Nanjing Agricultural University, Nanjing, China; 2 College of Animal Science and Technology, Jilin Agricultural University, Changchun, China; 3 School of Ecology and Environment, Northwestern Polytechnical University, Xi’an, China; 4 Shanghai BIOZERON Biotechnology Company Ltd, Shanghai, China; 5 Department of Special Economic Animal Nutrition and Feed Science, Institute of Special Animal and Plant Sciences, Chinese Academy of Agricultural Sciences, Changchun, China; 6 Buffalo Research Institute, Chinese Academy of Agricultural Sciences, Nanning, China; 7 CAS Key Laboratory for Agro-Ecological Processes in Subtropical Region, Institute of Subtropical Agriculture, Chinese Academy of Sciences, Changsha, China; 8 Biomedicine Discovery Institute, Department of Microbiology, Monash University, Clayton, Australia; 9 Section for Computational and RNA Biology, Department of Biology, University of Copenhagen, Copenhagen, Denmark; 10 Department of Agricultural, Food and Nutritional Science, University of Alberta, Edmonton, Canada; 11 Faculty of Biosciences, Norwegian University of Life Sciences, Aas, Norway

**Keywords:** Ruminant, Gastrointestinal microbiome, Metagenome-assembled genomes, Alphaproteobacteria, Feed efficiency

## Abstract

**Background:**

Gastrointestinal tract (GIT) microbiomes in ruminants play major roles in host health and thus animal production. However, we lack an integrated understanding of microbial community structure and function as prior studies. are predominantly biased towards the rumen. Therefore, to acquire a microbiota inventory of the discrete GIT compartments, In this study, we used shotgun metagenomics to profile the microbiota of 370 samples that represent 10 GIT regions of seven ruminant species.

**Results:**

Our analyses reconstructed a GIT microbial reference catalog with > 154 million nonredundant genes and identified 8745 uncultured candidate species from over 10,000 metagenome-assembled genomes. The integrated gene catalog across the GIT regions demonstrates spatial associations between the microbiome and physiological adaptations, and 8745 newly characterized genomes substantially expand the genomic landscape of ruminant microbiota, particularly those from the lower gut. This substantially expands the previously known set of endogenous microbial diversity and the taxonomic classification rate of the GIT microbiome. These candidate species encode hundreds of enzymes and novel biosynthetic gene clusters that improve our understanding concerning methane production and feed efficiency in ruminants. Overall, this study expands the characterization of the ruminant GIT microbiota at unprecedented spatial resolution and offers clues for improving ruminant livestock production in the future.

**Conclusions:**

Having access to a comprehensive gene catalog and collections of microbial genomes provides the ability to perform efficiently genome-based analysis to achieve a detailed classification of GIT microbial ecosystem composition. Our study will bring unprecedented power in future association studies to investigate the impact of the GIT microbiota in ruminant health and production.

**Video abstract**

**Supplementary Information:**

The online version contains supplementary material available at 10.1186/s40168-021-01078-x.

## Background

The history of ruminant livestock is tightly interwoven with that of humans due to their capability to convert fibrous plant substrates into accessible nutrients including meat and milk [1]. Gastrointestinal tract (GIT) microbial communities, especially those of the rumen microbiome, are believed to play an important role in such energy conversion and also their overall performance [2]. In this regard, the microbiomes of ruminant livestock are increasingly studied with many thousands of metagenome-assembled genomes (MAGs) obtained from dairy cows, sheep, and deer with a focus on the rumen microbiome [3]. However, the microbiomes across all GIT in yak, buffalo, and goat, which are also agriculturally important ruminant species, remain largely unstudied.

Ruminants are a mammalian lineage that exhibit substantial morphological and ecological diversity and have evolved over the last 40 million years to include both grazers and browsers [4]. These lineages have adapted to diverse habitats, spanning mesic environments to high altitude extremes, and as a result, they consume a diverse range of vegetation resulting in various strategies of dietary fiber digestion and nutrient harvesting [5]. Thus, it is expected that the diverse ruminant lineages possess distinct GIT microbiomes given their major variations in diet, as well as morphology, physiology, and behavior variations in the diet and their differences in morphological, physiological, and behavioral characteristics [6, 7]. This is consistent with a recent study by Glendinning et al. [3], in which they observed significant differences between ruminal microbiomes, as measured by taxonomic composition, carbohydrate-active enzyme genes, and KEGG orthologs, between cows, sheep, reindeer, and red deer. Consequently, characterizing the GIT microbiomes from a diverse array of host species is critical to developing a fundamental understanding of the structure and function of ruminant microbial communities, which will ultimately facilitate the knowledge-based development of sustainable ruminant production by increasing feed efficiency and general health.

Unlike swine, poultry, and humans, ruminants have evolved a compartmentalized stomach with four chambers (rumen, reticulum, omasum, and abomasum) in their GIT. The rumen hosts a wide spectrum of microbes that play key roles in plant processing, including the production of both energy precursors to fuel their ruminant hosts (e.g., volatile fatty acids) as well as greenhouse gases that cause global climate change (e.g., methane) [8, 9]. The taxonomic profiles and associated functions of the microbes inhabiting the rumen have been extensively studied in the past decade [9–13], and the fundamental knowledge acquired has facilitated regulation of rumen fermentation [14], development of biofuels [10, 15], improvement in feed efficiency [16], and reduction of enteric methane emissions [17, 18]. However, our knowledge of these processes is relatively incomplete, as the complete ruminant GIT contains 10 distinct physical compartments (rumen, reticulum, omasum, abomasum, duodenum, jejunum, ileum, cecum, colon, and rectum), and each region is spatially specialized depending on factors including physiology substrate availabilities, retention time of digesta, and pH levels [8, 19]. These factors are all expected to have a profound impact on the local microbial assemblages and functions, thereby affecting the digestive, immunological, metabolic, and endocrinological processes in ruminants [20]. Thus, a detailed mapping and characterization of the microbiomes in all GIT regions is required to gain a comprehensive understanding of the GIT microbiome’s roles in ruminant biology.

Here, we generated a ruminant GIT microbial gene catalog that comprises 154,335,274 genes and built genome compendia from 370 GIT content samples, spanning 10 different GIT regions sampled from seven ruminant species (dairy cattle, *Bos taurus*; water buffalo, *Bubalus bubalis*; yak, *Bos grunniens*; goat, *Capra aegagrus*; sheep, *Ovis aries*; roe deer, *Capreolus pygargus*; water deer, *Hydropotes inermis*). In addition, we assembled 10,373 metagenome-assembled genomes (MAGs), including potentially 8,745 novel uncultured bacterial and archaeal species. The findings greatly expand our understanding of the GIT symbiotic microbiome in ruminants and provide new insights for investigating the GIT microbiome’s role in host health and production.

## Results and discussion

### A microbial reference gene catalog of the ruminant GIT

We collected 370 content samples from 10 GIT regions, including the stomach (rumen, reticulum, omasum, and abomasum; *n* = 148), small intestine (duodenum, jejunum, and ileum; *n* = 111), and large intestine (cecum, colon and rectum; *n* = 111) of seven ruminant species (dairy cattle, water buffalo, yak, goat, sheep, roe deer, and water deer) (Additional file 2: Table S1). We performed shotgun metagenomic sequencing of genomic DNA extracted from these samples and obtained a total of 9.8 terabytes (Tb) of Illumina sequence data (Additional file 2: Table S2). After quality control of the data, 6.5 Tb of sequence data remained for the subsequent analyses (“Methods”; Additional file 1: Fig. S1, S2).

**Fig. 1:**
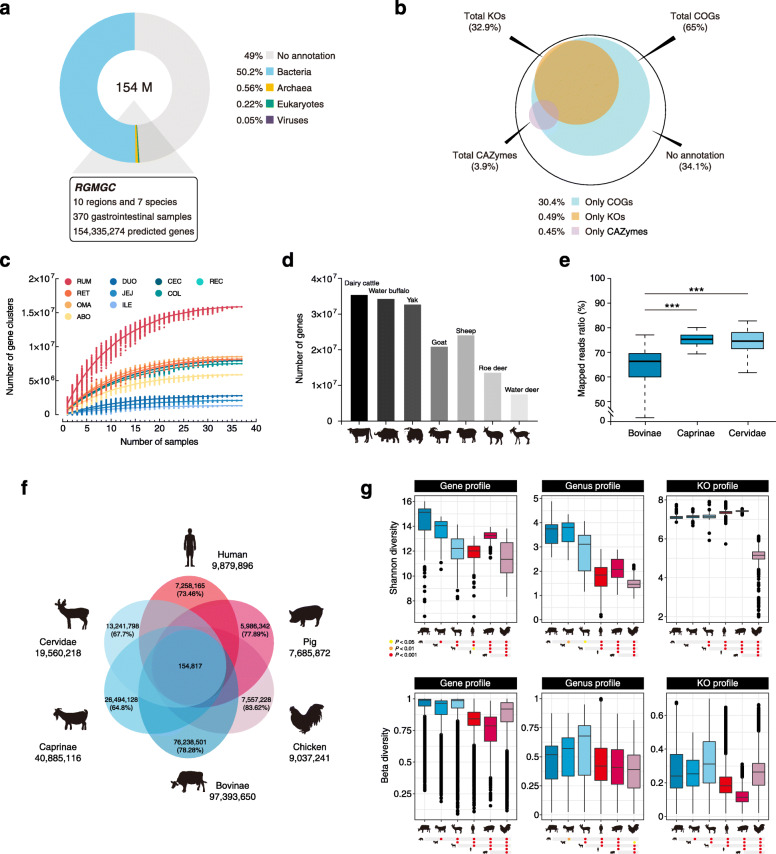
Ruminant GIT microbial reference gene catalog. **a** Breakdown of taxonomic annotations for the RGMGC. **b** The RGMGC was annotated according to three functional categories (COGs, KOs, and CAZymes). Percentages of identified genes in the specified functional categories are shown. **c** Accumulation curve depicting numbers of nonredundant gene clusters against numbers of investigated samples from different regions. RUM, rumen; RET, reticulum; OMA, omasum; ABO, abomasum; DUO, duodenum; JEJ, jejunum; ILE, ileum; CEC, cecum; COL, colon; REC, rectum. **d** Samples of each species were clustered to yield a set of corresponding gene catalogs. **e** Percentage of total reads in each sample of the three ruminant families that could be mapped to the RGMGC. **f** Venn diagram of unique and shared genes between ruminant and monogastric animal catalogs. **g** Alpha diversity (Shannon index) and beta diversity at the gene, genus, and KO function levels. Data are shown as box plots. The horizontal lines indicate the medians, and the whiskers indicate the lowest and highest points within 1.5× the interquartile ranges into the lower and upper quartiles, respectively. Colored circles at the bottom indicate significance based on the relative index of each cohort according to the Wilcoxon rank-sum test. *P < 0.05, **P < 0.01, ***P < 0.001

We generated a total of 249.2 million (M) contigs and 469.7 M open reading frames (ORFs) via metagenomic assembly and ORF prediction (Additional file 1: Fig. S1). The ORFs covered 90% of the contigs, and 32.2% of the genes were identified as complete (Additional file 1: Fig. S3; Additional file 2: Table S2). The coverage of ORFs and completeness of predicted genes are comparable to the results of the human gut microbiome (86.7% and 33.3%, respectively) [21]. After clustering at 95% nucleotide sequence identity [22], we obtained a nonredundant ruminant GIT microbial gene catalog (RGMGC) with 154,335,274 genes (average length, 650 bp; Fig. 1a, b; Additional file 2: Table S3). Rarefaction curves approached asymptotes across all ruminant species with an average of 83.2% cumulative coverage among 10 GIT regions (Fig. 1c), indicating that these genes encompassed most of those encoded by the microbial taxa in these ruminant GITs. However, according to currently available databases, only 51% of the genes in the RGMGC were taxonomically classified as originating from bacteria (50.2%), archaea (0.56%), eukaryote (0.23%) and virus (0.05%; Fig. 1a), and 65% (100,323,994), 32.9% (50,732,442) and 3.9% (6,032,484; Fig. 1b) were annotated to cluster of orthologous groups of protein (COGs), KEGG orthologous groups (KOs) and carbohydrate-active enzymes (CAZymes), respectively. These results suggest that the RGMGC includes many unknown genes representing a highly complex taxonomic assemblage.

We compared our gene catalog to the previously published rumen metagenomic datasets generated by Hess et al. (2.5 M genes) [15] and Li et al. (13.8 M genes) [13] and found only 12.8% of the genes in the RGMGC overlapped either with one or both of the two sets (Additional file 1: Fig. S4a). In addition, we compared the RGMGC to the recently published large protein database (9.45 M protein clusters) for rumen microbiomes by Stewart et al. [12], and the proportion of overlap in the RGMGC was 6.8% (Additional file 1: Fig. S4b), indicating our RGMGC contains a large number of novel genes. We further aligned the published ruminant GIT metagenomic samples (*n* = 635, from 16 studies and ~ 11.3 Tb in total; Additional file 2: Table S4) to the RGMGC and found that approximately 82.5% of the quality-filtered reads were aligned (Additional file 2: Table S5), suggesting unprecedented coverage. We also found that 17.8 M of genes in RGMGC were not covered by these sequencing reads, of which 87.1% were assembled from the rest of non-rumen GIT regions (Additional file 2: Table S5), indicating the importance and uniqueness of the genes assembled from previously overlooked GIT regions. Collectively, this indicates that the RGMGC is the largest gene catalog for the ruminant GIT microbiome to date and thus will serve as an essential reference and baseline for further investigation of the symbiotic microbiome in ruminants.

### Variation and features of the ruminant GIT microbiome

To examine the association between functional and compositional features of the microbial communities and the host species, we constructed a representative GIT gene catalog for each of the seven focal ruminant species, spanning 8 to 36 M genes (for water deer and dairy cattle, respectively; “Methods” and Fig. 1d). The percentage of quality-filtered reads that mapped to the RGMGC was significantly lower for samples from the Bovinae (63.8%) than corresponding numbers from the Caprinae (75%) and Cervidae (71.7%) (Wilcoxon rank-sum test, *P* < 0.001; Fig. 1e). Although animal individuals, diet, age, and sex affected the GIT microbiome, consistent patterns were observed from analyses of samples from all seven included species (Additional file 1: Fig. S5), suggesting that members of the Bovinae have a much more complex GIT microbiome than the other species, which we hypothesize likely relates to their dietary diversity [4].

We further compared the present gut microbial gene catalog with that of human (*Homo sapiens*, 9.9 M) [22], and pig (*Sus scrofa domesticus*, 7.7 M) [23], which were mainly based on fecal samples, and chicken (*Gallus gallus domesticus*, 9.04 M) based on the content samples from different intestinal compartments [24]. We found that the RGMGC comprised of more predicted genes than these three available catalogs for monogastric animals (Fig. 1f), and the ruminant GIT pertains relatively to a higher variance, as measured by alpha and beta diversity, than that of monogastric animals at both gene taxonomic and functional levels (Fig. 1g; Additional file 2: Table S6, S7). These results are consistent with previous finding observations that ruminants harbor or contain a more complex microbial community than monogastric animals, and herbivores have a higher bacterial diversity and richness than omnivores [6]. However, we could not exclude the influence of sequencing depth in the capture of microbial genes because the given different sources were used in these studies.

### Regional organization and functional potentials of the ruminant microbiome

**Fig. 2:**
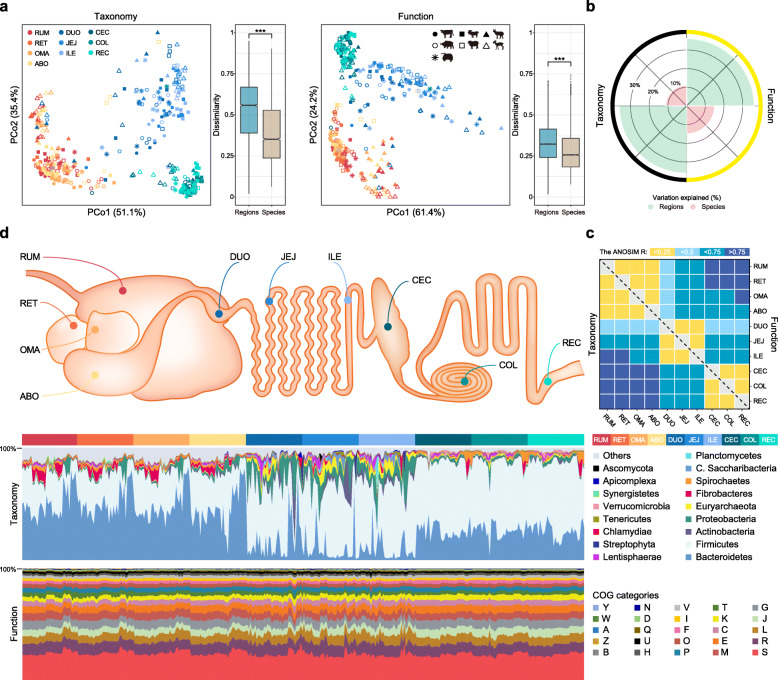
GIT functional and taxonomic variability in ruminants. **a** PCoA plot based on the relative abundances of genera and COGs. The colors and shapes of the symbols indicate regions and species, respectively. Bray-Curtis distances associated with regions and species are shown as box plots (Wilcoxon rank-sum test; ***P < 0.001). The horizontal lines indicate medians, and the whiskers indicate the lowest and highest points within 1.5× the interquartile ranges of the lower and upper quartiles, respectively. RUM, rumen; RET, reticulum; OMA, omasum; ABO, abomasum; DUO, duodenum; JEJ, jejunum; ILE, ileum; CEC, cecum; COL, colon; REC, rectum. **b** Variability in taxonomic and functional differences explained by regions and species. **c** Bray-Curtis dissimilarities were assessed by analysis of similarity (ANOSIM). **d** Relative abundances of major phyla and COG categories across GIT samples. A, RNA processing and modification; B, chromatin structure and dynamics; C, energy production and conversion; D, cell cycle control, cell division, chromosome partitioning; E, amino acid transport and metabolism; F, nucleotide transport and metabolism; G, carbohydrate transport and metabolism; H, coenzyme transport and metabolism; I, lipid transport and metabolism; J, translation, ribosomal structure, and biogenesis; K, transcription; L, replication, recombination and repair; M, cell wall/membrane/envelope biogenesis; N, cell motility; O, posttranslational modification, protein turnover, chaperones; P, inorganic ion transport and metabolism; Q, secondary metabolites biosynthesis, transport, and catabolism; R, general function prediction only; S, function unknown; T, signal transduction mechanisms; U, intracellular trafficking, secretion, and vesicular transport; V, defense mechanisms; W, extracellular structures; Y, nuclear structure; Z, cytoskeleton

To illustrate the regional organization of microbial communities and their associated functional potentials along the ruminant GIT, we first demonstrated that the microbiome primarily partitioned into three distinct GIT compartment groups (stomach, small intestine, and large intestine) at both the taxonomic and functional levels (Fig. 2a). Notably, the GIT regions accounted for much more of the variance detected (36%) than the species sampled (13%) (Fig. 2b). The analysis also revealed dramatic changes in microbial taxa across the 10 GIT regions (Fig. 2c, d). For example, *Prevotella* spp. and *Fibrobacter* spp. were dominant in the stomach region; *Bacteroides* spp., *Clostridium* spp., *Alistipes* spp., and *Ruminococcus* spp. were more prevalent in the large intestine; and *Escherichia* spp. had relatively high relative abundance in the small intestine (Additional file 1: Fig. S6; Additional file 2: Table S8). Interestingly, *Methanobrevibacter* spp. were much more abundant in the small intestine (3.7% of total microbial abundance) than in the stomach (0.71%) and large intestine (1.1%), indicating that these previously neglected methanogens may play a role in the maintenance of microbial functional homeostasis and anaerobic digestion in the small intestine. Together, these results suggest that GIT regions impose substantial local selection on the microbial community.

**Fig. 3:**
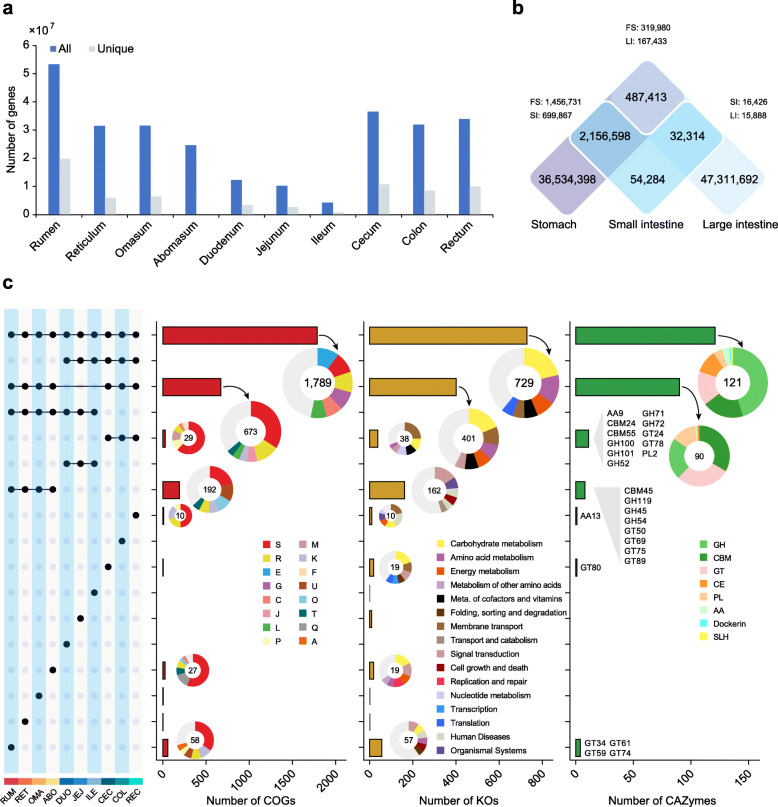
Specific functional features of the ruminant GIT microbiome. **a** All the nonredundant microbial genes across the ruminant GIT microbiome, with unique counts in each region. **b** Venn diagram of the unique and shared gene counts between the stomach (FS: rumen, reticulum, omasum, and abomasum), small intestine (SI: duodenum, jejunum, and ileum), and large intestine (LI: cecum, colon, and rectum). **c** Comparison of the levels of functional modules (COGs, KOs, and CAZymes) of the microbiome across regions of the ruminant GIT. The left panel shows sets included in the intersection and independent sites, and the right bar or pie charts show the categories of the functional modules in these sets. The major enriched categories are shown in the legend. A, RNA processing and modification; C, energy production and conversion; E, amino acid transport and metabolism; F, nucleotide transport and metabolism; G, carbohydrate transport and metabolism; J, translation, ribosomal structure, and biogenesis; K, transcription; L, replication, recombination and repair; M, cell wall/membrane/envelope biogenesis; O, posttranslational modification, protein turnover, chaperones; P, inorganic ion transport and metabolism; Q, secondary metabolites biosynthesis, transport, and catabolism; R, general function prediction only; S, function unknown; T, signal transduction mechanisms; U, intracellular trafficking, secretion, and vesicular transport. GH, glycoside hydrolases; GT, glycosyl transferases; CE, carbohydrate esterases; PL, polysaccharide lyases; CBM, carbohydrate-binding modules; SLH, S-layer homology module; AA, auxiliary activities

To further characterize the regional variation of microbial function, we compared the gene functions from the 10 GIT regions (Additional file 1: Fig. S7; Additional file 3: Table S9) and observed a decreasing trend in the number of predicted genes from the rumen (53 M) to the small intestine (4 to 12 M), followed by an increase in the large intestine (32 to 36 M). Similar patterns were observed in regional unique genes and alpha diversity (Fig. 3a, b; Additional file 1: Fig. S8). These results further confirm the microbial contribution of the large intestine in digestion. The lower number of genes and microbial diversity in the small intestine is likely related to the overall lower biomass in this structure due to the short transit time of the digesta within it, together with intermittent food substrate delivery, the influx of digestive enzymes and bile acid, and physicochemical factors including mucus thickness, host-derived antimicrobials, pH levels, and oxygen concentrations [2, 20].

When exploring the abundance of COG functions among the GIT regional microbiomes, we found that COGs associated with degradation of plant carbohydrates, dietary proteins, and lipids were enriched in the stomach, nucleic acid metabolism in the small intestine, and protein synthesis in the large intestine, respectively (Additional file 3: Table S9; Additional file 4: Table S10). Further, we observed a high representation of KOs involved in carbohydrate metabolic pathways in both the stomach and large intestine microbiome (Fig. 3c; Additional file 4: Table S11), and 33.3% of the shared CAZymes between these two regions were assigned to carbohydrate-binding module families (Additional file 4: Table S12). These results suggest the GIT microbiome has substantial regional functional heterogeneity. Notably, we found regional differences in the functions of microbial membrane transport systems (mainly ABC transporters; Additional file 4: Table S11) associated with the transport of carbon and nitrogen nutrients. For example, microbial transport proteins in the stomach seem to be particularly responsible for transporting alpha-glucoside, putrescine, and urea, while those in the large intestine are particularly associated with the transport of monosaccharides such as N-acetylglucosamine. Moreover, we found that five specific membrane transport proteins in the jejunum microbiome were associated with general L-amino acid and dipeptide transport. These results underlie the regional differentiation in nutrient utilization, highlighting the need for systematic consideration of the entire digestive tract in ruminant nutrition research.

### Reconstructing 10,373 metagenome-assembled genomes from the ruminant GIT

To further explore the ruminant GIT microbiota at the genome level, we performed contig binning (“Methods”; Additional file 1: Fig. S1) and generated 116,138 bins. After quality evaluation (i.e., genome completeness and contamination) using CheckM [25], we obtained 28,543 MAGs that met or exceeded medium quality (≥ 50% completeness and < 10% contamination) and could be resolved to bacterial or archaeal lineages (Additional file 1: Fig. S9; Additional file 4: Table S13). We also explored how many of these bins represented known eukaryotes or viral sequences and found that only eight bins had ≥ 50% of their genome aligned to a protozoan organism and 310,661 viral contigs were detected with ≥ 5 kilobases (kb) length (Additional file 4: Table S14, S15). Because of the lack of any complete eukaryote or virus genome in the ruminant GIT, we focused on the 28,543 MAGs resolved to a prokaryotic lineage.

We applied a dereplication pipeline at ≥ 99% average nucleotide identity (ANI) and generated 10,373 nonredundant MAGs. Of these, 2211 were estimated to be near-complete (> 90% completeness and < 5% contamination) and 8162 were medium-quality (quality scores [26]: 4,852 > 50 and 3,310 ≤ 50) (Additional file 4: Table S16). These 10,373 MAGs ranged in size from 418.3 kb to 9.88 megabases (Mb), with N50 values ranging from 1.9 kb to 1.04 Mb (Additional file 4: Table S16). Using the Genome Taxonomy Database [27], 10,213 MAGs were assigned to bacterial phyla and 160 MAGs were assigned to archaeal orders (Additional file 4: Table S16).

**Fig. 4:**
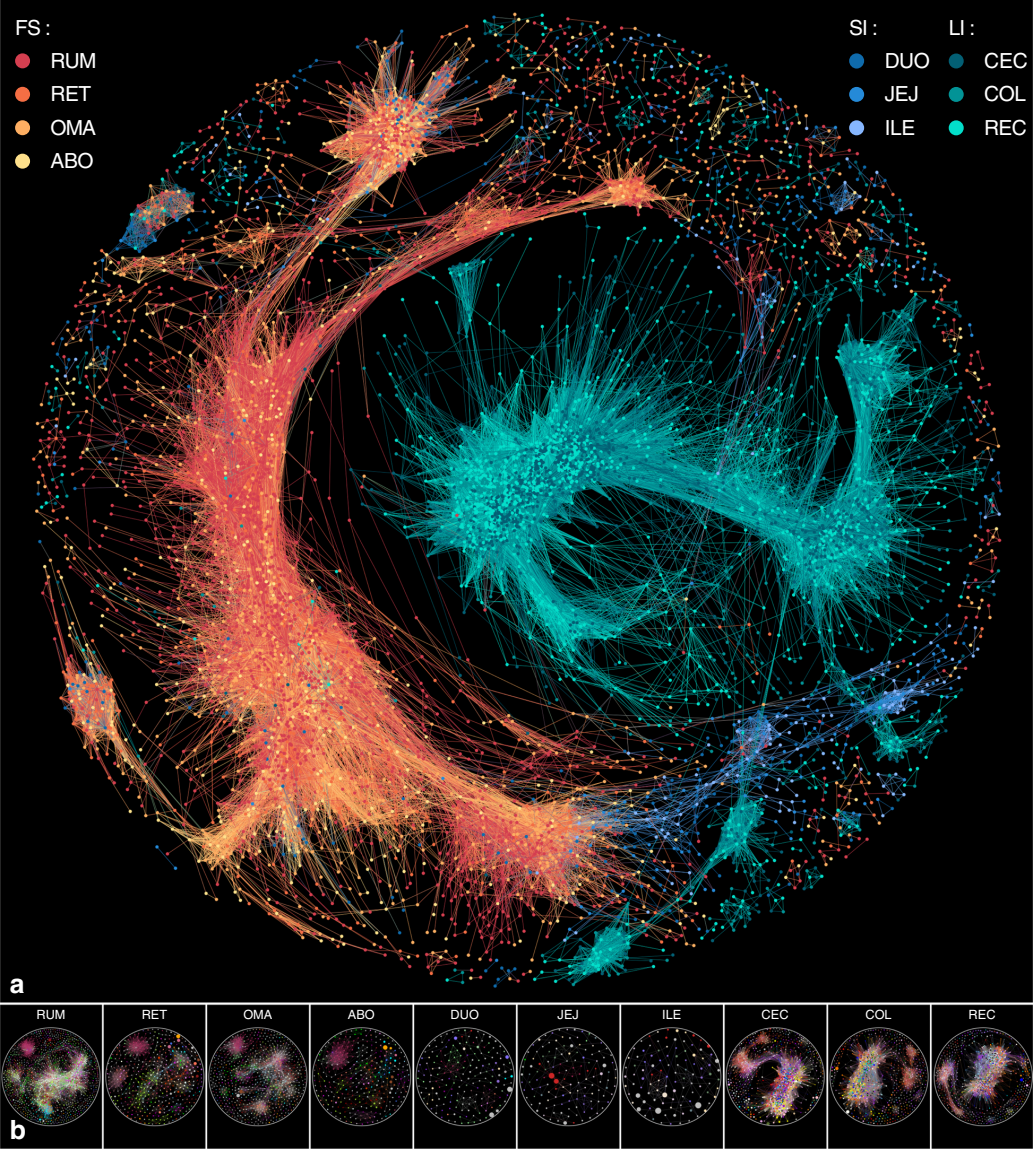
Maps of the GIT microbial interaction network in ruminants. **a** Co-occurrence interaction network of the 10,373 MAGs based on Spearman correlation indices calculated from the abundances of MAGs in each sample (connections indicate relationships with |Spearman’s rank correlation coefficient| > 0.85 and *P* < 0.01). The colors of the nodes indicate sites, and edges are colored according to the nodes. **b** MAGs localized within the 82 clustered modules are indicated with different node colors. The size of each node represents the average abundance of the MAGs in the indicated sites. FS, stomach; SI, small intestine; LI, large intestine. RUM, rumen; RET, reticulum; OMA, omasum; ABO, abomasum; DUO, duodenum; JEJ, jejunum; ILE, ileum; CEC, cecum; COL, colon; REC, rectum

We found that these MAGs also divided into three distinct regional groups (Additional file 1: Fig. S10, S11), indicating the distributive heterogeneity of the microbiome at the single genome level. Then we evaluated whether this difference was reflected by distinct co-associations between taxa at each GIT region. We observed higher clustering coefficients of co-associated MAG taxa in the stomach and large intestine (average degree = 12.6 and 80.2, respectively), and a lower low clustering coefficient of MAG taxa present in the small intestine (average degree = 2.6) (Fig. 4a), suggesting a specialized spatial co-association among MAGs in individual GIT regions. We further explored individual taxa with high regional specificity or strong spatial patterns by clustering the overall network into 82 modules and found that module 47 containing 367 MAGs (maroon nodes in Fig. 4b) was only present in the stomach and small intestine. Taxonomic assignment revealed that they were mainly classified into the genus *Prevotella* (11.7%; Additional file 4: Table S17). Further analysis of the genome properties (GPs) found that functions related to amino acid biosynthesis and catabolism were enriched in these MAGs (Additional file 4: Table S17), which is likely associated with the distinct metabolism in these regions. These results suggest that regional nutritional factors strongly influence the local spatial structuring of the microbiota. We further examined the aggregation degree of networks among the GIT regions and found that hub MAGs in the large intestine were mainly from the genus CAG-110 (Firmicutes bacterium; Additional file 4: Table S18). Comparative genome analyses revealed a general lack of GPs associated with ethanolamine degradation and utilization in CAG-110 genomes from the stomach and small intestine, whereas these GPs were abundant in large intestine CAG-110s (86% of the total genomes; Additional file 1: Fig. S12). Ethanolamine is mainly derived from the phospholipid component of the intestinal epithelium, which can be degraded into acetaldehyde and ammonia [28], thus providing a carbon and nitrogen source for microbes in the large intestine. This finding suggests that host-derived factors also influence distributions of individual taxa in GIT regions.

### Compendium of 8745 unknown species-level genomes

To elucidate whether these MAGs represented novel taxa, we compared the 10,373 MAGs to 43,532 prokaryotic genomes in the GenBank database and a collection of published ruminant microbial genomes (RMG, *n* = 7,052) that includes a recent rumen MAG dataset by Stewart et al. [11, 12] (Additional file 4: Table S19; Additional file 4: Table S20). Using species-level thresholds (≥ 95% ANI and ≥ 60% alignment) [29], 8745 of the MAGs did not match any available reference genomes. Of these, 1886 were near-complete genomes, while the remaining 6859 were medium-quality, representing unknown genomes characterized at the species level (USGs; Additional file 1: Fig. S13; Additional file 4: Table S21). The highest proportion (48.3%) of USGs were retrieved from the large intestine, followed by stomach (41.9%) and small intestine (9.8%) samples (Additional file 1: Fig. S14). These 8745 USGs were assigned to 28 phyla, 89 orders, 162 families, and 382 genera, and 36% of these USGs could not be classified to a known genus (Additional file 4: Table S21), meaning that a substantial portion of the USGs likely represent novel genera. Prevalent USGs classified at the order level were Bacteroidales (24.2%), Oscillospirales (24.2%), and Lachnospirales (10.1%), while the top genera all belonged to the order Bacteroidales, including RC9 (3.3%), *Alistipes* (3.2%), and *Prevotella* (2.6%) (Additional file 1: Fig. S15). These results suggest that the three common orders still contain considerable uncultured diversity in the ruminant GIT.

**Fig. 5:**
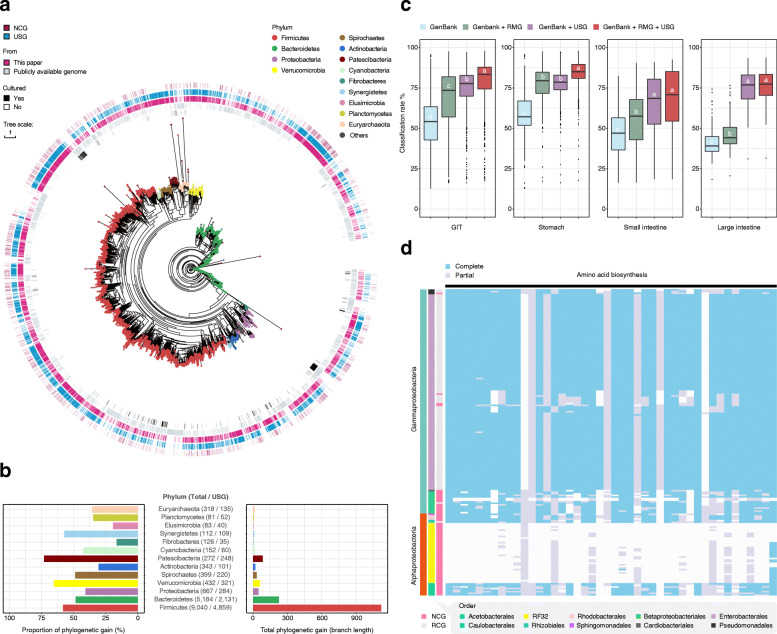
Novel species have distinct functional capacities. **a** The maximum-likelihood tree of the 10,373 MAGs identified in this study and previously published ruminant microbial genomes (17,425 genomes in total) was produced from concatenated protein sequences using PhyloPhlAn [30]. Clades are colored according to phyla. Genome information is presented in the outer layers. NCG, near-complete USGs. **b** The phylogenetic gain contributed by the microbial tree of the ruminant GIT provided by the USGs is shown as proportional increases in branch length per phylum (left) and absolute branch lengths (right). Numbers of USGs are indicated in parentheses (left, total; right, USGs). **c** Comparison of the read classification rates of all the ruminant GIT metagenomic samples using the following datasets: a common database consisting of all complete microbial genomes in GenBank, the GenBank database plus the RMG, the GenBank database plus the USGs identified in this study, and all three datasets. The Wilcoxon rank-sum test was used to assess the differences, and significant differences (*P* < 0.05) are indicated by different letters (a, b, c, and d). **d** Comparison of functional differences in genome properties between the NCG (*n* = 69) and RCG (*n* = 125) sets of Proteobacteria. The “Complete” and “Partial” heatmaps indicate functional profiles of GPs of the genomes involved in amino acid biosynthesis

To understand the phylogenetic position of uncultured species in the ruminant GIT, we then placed these USGs in a taxonomic framework based on entries from published ruminant microbial genomes (Additional file 4: Table S20). A maximum-likelihood phylogeny was built on the basis of the conserved proteins determined using PhyloPhlAn [30] and showed that the USGs covered > 75% of the total phylogenetic diversity and provided on average 56.2 and 35.8% improvements in phylogenetic diversity for ruminant bacterial and archaeal lineages, respectively (Fig. 5a, b). To further evaluate the improvement by USGs for taxonomic classification of ruminant microbiota, we profiled 635 published ruminant metagenomic datasets (Additional file 2: Table S4) alongside our 370 new datasets, by using a combination of GenBank entries, RMG entries, and USGs. Using these genomes, we observed an average 80% taxonomic classification rate of reads across these ruminant metagenomic data (Fig. 5c), which is higher than a previous rate of 70% for rumen metagenomic datasets [12]. Strikingly, the USGs provided a 10.8% improvement for these data compared to the use of GenBank plus RMG database (Additional file 4: Table S22). The USGs also provided improvements in a read classification rate of 5.7, 11.4, and 30.4% (to 83.5, 67.3, and 76.8%, respectively) for the stomach, small intestine, and large intestine, respectively (Additional file 4: Table S22). Notably, the classification rate by using GenBank plus USG for the rumen was 75.9%, a result which is comparable to that with the GenBank plus RMG database (78.6%; Additional file 4: Table S22), indicating the power of our multi-species and high-depth sequencing in taxonomic profiling even for rumen microbiome which has been extensively examined recently.

### Functional repertoire of the USGs

CAZymes such as cellulase, hemicellulases, oligosaccharide-degrading enzymes, and polysaccharide utilization loci (PUL) collectively play important roles in carbohydrate utilization in the ruminant GIT [31]. We identified 850,749 CAZyme-predicted proteins and 12,578 PULs from these 8745 USGs (Additional file 4: Table S23; Additional file 4: Table S24). In addition, to further explore the metabolic potential of these USGs, we screened for the presence of biosynthetic gene clusters (BGCs) that encode secondary metabolites. A total of 12,006 BGCs were detected from 5836 USGs, which were divided into 32 different products, and 87.8% of these BGCs represented novel clusters (Additional file 1: Fig. S16; Additional file 4: Table S25). The identification of these novel USGs will facilitate improvement in understanding carbohydrate metabolism in the ruminant GIT and provide a rich source of novel bioactive compounds with potential pharmaceutical applications, such as antibiotics.

We further compared the 1886 near-complete USGs with cultured isolates including the Hungate1000 project data [10] (Additional file 4: Table S20), hereafter referred to as the ruminant cultured genomes (RCGs). Principal coordinates analysis (PCoA) revealed a clear difference in the functional repertoires of GPs between the USGs and RCGs, which is mostly explained by the phylum Proteobacteria (ANOSIM, *R* = 0.833, *P* = 0.001; Additional file 1: Fig. S17, S18). Interestingly, RCGs are dominated by the class Gammaproteobacteria (99.2% of Proteobacteria RCGs), while USGs are mainly composed of Alphaproteobacteria (73.9% of Proteobacteria USGs) (Fig. 5d). Given the members of Proteobacteria tend to be associated with significant functional variability in the gut microbiome [32], we then compared GPs in the genomes assigned to these two classes and detected 671 differentially represented GPs (Chi-squared test, adjusted *P* < 0.05; Additional file 4: Table S26). We next found that the genes encoding two types of aminoacyl-tRNA synthetases (glutaminyl-tRNA and asparaginyl-tRNA synthetase) were widespread in Gammaproteobacteria genomes (Additional file 1: Fig. S19). In contrast, most Alphaproteobacteria genomes lacked these genes, but contained heterotrimeric GatABC, which provides an alternative to direct aminoacylation [33]. These results suggest that microbes of the class Alphaproteobacteria may have evolved to have specific metabolic or survival capabilities in the ruminant GIT. This inference was corroborated by analysis of the high-quality, but reduced genomes of USGs (RF32 order; *n* = 38, genome size = 1.59 Mb), which revealed substantial losses of GPs involved in amino acid biosynthesis (Fig. 5d; Additional file 4: Table S26). Together, these findings may offer clues for improving cultivation strategies in the future.

### Novel findings on ruminant GIT methanogenic archaea and hydrogen metabolism

**Fig. 6:**
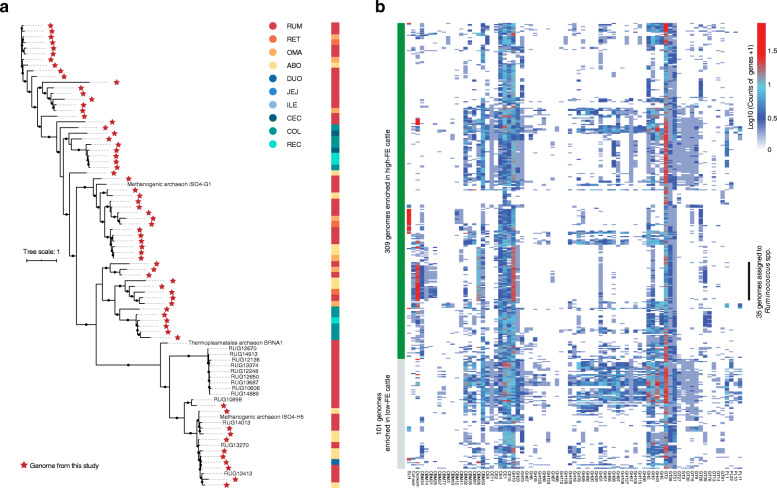
Phylogenetic tree of order Methanomassiliicoccales and analysis of FE-enriched genomes. **a** Maximum-likelihood tree of the 82 Methanomassiliicoccales genomes constructed using PhyloPhlAn [30]. Stars indicate the 66 MAGs reconstructed in this study. The colored bars in the outer layer indicate the GIT regions from which these genomes were obtained these genomes. RUM, rumen; RET, reticulum; OMA, omasum; ABO, abomasum; DUO, duodenum; JEJ, jejunum; ILE, ileum; CEC, cecum; COL, colon; REC, rectum. **b** Heatmaps showing counts of selected CAZyme-encoding genes in the 410 differentially enriched genomes identified between the high- and low-FE groups. The most prevalent assigned genera in these genomes are denoted on the left

Although ruminant GIT methanogens are major sources of anthropogenic methane emissions, the methanogenic archaea of ruminants are still poorly characterized. We retrieved a total of 135 USGs from 160 archaeal MAGs across the GIT regions (Additional file 1: Fig. S20; Additional file 4: Table S27), including 56 USGs assigned to Methanomassiliicoccales (formerly called “Rumen Cluster C”; Fig. 6a), forming a core group of methylotrophic methanogens in the rumen [34]. Previously only three complete genomes were available from the rumen environment. Thus, these new archaeal genomes can enhance the discovery power of metagenomics, by identifying novel lineages and aiding the selection of targets for in-depth analyses.

Hydrogen is primarily produced through microbial fermentation processes and can be the major energy source for methanogens. This process is supported by hydrogenases that catalyze H_2_ production or consumption [18]. Thus, we further focused on the organisms possessing hydrogenases (NiFe-, FeFe-, and Fe-hydrogenases) among the 10,373 MAGs, and found that the hydrogenases-encoding MAGs (*n* = 6,152) were distributed across 24 phyla, 71 orders, and 304 genera (Additional file 1: Fig. S21; Additional file 4: Table S28). Of these 6,152 MAGs, 3003 encoded enzymes for fermentative H_2_ production (Firmicutes = 72.7%; Bacteroidetes = 14.5%), whereas 95 MAGs encoded H_2_-uptake hydrogenases and the methyl-CoM reductases as the terminology is a little loose. (*mcrA* genes) related to hydrogenotrophic methanogenesis (e.g., *Methanobrevibacter* spp.) (Additional file 5: Table S29). We also found 352 MAGs that encoded both hydrogenases and the required terminal reductases in pathways that can potentially inhibit methane production by redirecting H_2_ uptake away from methanogenesis. These included MAGs associated with acetogenesis (193 MAGs, e.g., *Eubacterium* spp. and *Ruminococcus* spp.), fumarate reduction (116, e.g., *Selenomonas* spp.), sulfidogenesis (47, e.g., *Desulfovibrio* spp.), and nitrate ammonification (49, e.g., *Campylobacter* spp.) (Additional file 5: Table S29). Overall, our findings of these methanogen lineages and subsequent implications for H_2_ metabolism and gastrointestinal methanogenesis may provide novel targets for mitigating enteric methanogenesis in ruminant production.

### Novel insights into cattle feed efficiency

Feed efficiency (FE) significantly affects ruminant productivity and is putatively influenced by variation in the rumen microbiome [16]. However, no significant differences in the structure and function of ruminant microbiota communities have been documented between low- and high-FE Angus beef cattle [35]. We re-analyzed the dataset from Li et al. [35] using both our USGs and the RMG datasets and found 5 and 11 significantly differing taxa between low- and high-FE cattle at the genus and species levels, respectively (*t*-test, *P* < 0.05; Additional file 6: Table S30), including *Pseudobutyrivibrio ruminis*, *Clostridium lavalense*, and *Pseudomonas aeruginosa*. Next, we compared the abundance of microbial genomes between the two groups and identified 309 and 101 enriched genomes in the high- and low-FE cattle, respectively (near-complete, log_2_^fold-change^ > 1 and *P* < 0.05; Fig. 6b). In the subsequent examination of metagenome-wide associations between these enriched genomes and FE traits, we detected 35 positively and two negatively correlated genomes with FE (|Pearson correlation coefficients| > 0.5 and *P* < 0.05; Additional file 1: Fig. S22). Interestingly, the genus *Ruminococcus* accounted for a large proportion of the FE-associated genomes. Previous studies demonstrated that *Ruminococcus* spp. play important roles in the degradation of carbohydrates [36]. Similarly, we found a high abundance of genes related to amylosome complexes, including GH13, CBM26, and dockerin in genomes of *Ruminococcus* spp. (Additional file 7: Table S31). These results suggest that enhanced degradation of plant polysaccharides might be related to high FE. Moreover, we found 5- and 11-fold enrichment in genomes assigned to *Sharpea azabuensis*, which has been shown to be associated with the metabolic pathway of lactate conversion to propionate and butyrate [37], in the high-FE cattle (Additional file 7: Table S31). Thus, enrichment of *S. azabuensis* in high-FE cattle may optimize ruminal fermentation and improve energy efficiency. These genome-derived findings will lay the foundation for future fundamental and practical studies.

## Conclusions

The ruminant gastrointestinal microbiome is far more complex and diverse than the microbiome of the human gut, though until now, comprehensive surveys of the microbial species and genes in the ruminant GIT remained limited. Mainly, researchers have been .focused on understanding the rumen microbiota's contribution to the host, environment, and humans in the last decade and neglect to understand the gastrointestinal microorganisms themselves. As a result, although there are some new techniques beyond the culturing studies that have been applied to rumen research, it is still difficult to precisely the composition and function of the GIT microbiome in ruminants.

Here, we subjected the GIT microbiomes of seven diverse ruminant livestock species to large-scale metagenomic sequencing and generated a catalog of genes with unprecedented coverage and resolution, enabling a comprehensive understanding of the ruminant GIT microbiota and providing detailed information on microbial genetic diversity that may facilitate future targeted analyses. Through this high-coverage GIT microbial gene catalog, we clear demonstrate spatial associations between both taxonomic and functional elements of the microbiome and physiological adaptations across the GIT regions. Through training on a large amount of published microbial datasets from ruminants, we validated the unprecedented coverage of this gene set in the ruminant GIT microbiome. The microbial gene catalog generated will be helpful for understanding functions of the ruminant GIT microbiome and its relationship with the host, especially in the lower gut, which is far more important for ruminants than previously appreciated [2]. In addition, we improved the classification of reads from the ruminant microbiome and assembled 10,373 MAGs from 10 GIT regions including 8745 novel USGs assigned to bacterial and archaeal lineages. Together, these genomes improve the read classification rate by 5.7, 11.4 and 30.4% for the stomach, small intestine, and large intestine, respectively, compared to the combination of GenBank entries and all RMG entries. Collectively, these newly characterized USGs substantially expand the genomic landscape of ruminant microbiota. Importantly, these USGs provide access to the uncultured microbial diversity and may offer clues for improving cultivation strategies and future manipulation of the ruminant GIT microbiome.

Currently, ruminant livestock production faces great challenges, driven by heightened awareness of global warming and climate change caused by greenhouse gases, such as methane. Our study represents an updated dataset of archaeal genomes and further extends the potential pathways of redirecting H_2_ uptake away from methanogenesis. Another effective strategy for methane mitigation is to increase the feed efficiency of ruminant livestock. Our updated dataset can substantially differentiate the structure and function of the ruminal microbiota community between high- and low-FE cattle, providing an important potential marker for breeding of high-FE cattle. The generated genomic resource will facilitate the understanding and investigation on the GIT microbiota that contribute to the efficient and sustainable production of ruminants.

## Methods

### Experimental design and sample collection

For the de novo generation of metagenomic sequencing data, 370 luminal digesta samples were collected from the GIT (the rumen, reticulum, omasum, and abomasum in the stomach; the duodenum, jejunum, and ileum in the small intestine; and the cecum, colon, and rectum in the large intestine) of seven ruminant species: dairy cattle (*n* = 6), water buffalo (*n* = 5), yak (*n* = 5), goat (*n* = 6), sheep (*n* = 5), roe deer (*n* = 5), and water deer (*n* = 5) (Additional file 2: Table S1). Animals were slaughtered three hours later following the morning feeding. To greatly minimize the potential contamination across GIT regions, animal carcasses were positioned in their ‘natural’ way without unnecessary moving for sampling. Next, GIT regions were tied off separately by using a thread and were subsequently transferred to sterilized brown paper. Luminal contents from each region were transferred to a sterile container for homogenization and then collected via DNase- and RNase-free tubes. All collected fresh samples were frozen in liquid nitrogen and transported to the laboratory in a dry-ice pack, where they were immediately stored at − 80 °C before total DNA was extracted.

### DNA extraction and metagenomic sequencing

DNA was extracted from each sample (~ 200 mg per sample) following the protocol from Yu and Morrison [38] based on repeated bead-beating using a mini-bead beater (Biospec Products, Bartlesville, USA). The DNA integrity was controlled by electrophoresis on 0.8% agarose gels, and the concentration and quality were determined using a Nanodrop ND-1000 (Thermo Scientific, Wilmington, USA). A metagenomic library with an insert size of 350 bp was constructed from high-quality DNA extracted from each sample using the TruSeq DNA PCR-Free Library Preparation Kit (Illumina, San Diego, CA, USA) following the manufacturer’s instructions and then sequenced on an Illumina NovaSeq platform. The sequencing generated a total of 9.8 Tb of Illumina data from the 370 samples and approximately 65.3 billion sequencing reads with a read length of 150 bp (Additional file 2: Table S2).

### Construction of the ruminant GIT microbial gene catalog

Adapters from the Illumina data were first trimmed using Trimmomatic [39] (v.0.33). Then, to decrease potential DNA contamination from the environment, we mapped the sequence data to host, plant (mainly from the animals’ diets and recruited the closest genomes from NCBI; Additional file 1: Fig. S1), and human genomes using BWA-MEM [40] (v.0.7.17) (Additional file 1: Fig. S2; Additional file 2: Table S2). A total of 6.5 Tb of sequencing reads remained, referred to as high-quality reads, including 1108.9 Gb for dairy cattle, 738.6 Gb for water buffalo, 715.8 Gb for yak, 1131.8 Gb for goat, 1048.9 Gb for sheep, 849.2 Gb for roe deer and 867.7 Gb for water deer. To construct a comprehensive ruminant GIT microbial gene catalog, we individually assembled the high-quality reads from each sample using MEGAHIT [41] (v.1.1.1, parameter: --min-contig-len 500 -t 40) and IDBA-UD [42] (v.1.1.3, parameter: --pre_correction --min_contig 500 --num_threads 40 --mink 90 --maxk 124). This resulted in 249,094,990 (average N50, 3,781 bp) and 15,398,581 (average N50, 4,212 bp) contigs longer than 500 bp from the two assemblies, respectively, and a total contig length of 361.2 × 10^9^ bp (Additional file 2: Table S2). Then, the contigs from the two approaches were combined using Minimus2 [43] (AMOS, v.3.1.0) with the parameters “-D CONSERR = 0 OVERLAP = 100 MINID = 100.” To reduce errors generated during the assembly, all reads were mapped back to the assembled contigs using BWA-ALN [44] (v.0.7.17) and SAMtools [45] (v.1.9), whereupon single bases, insertions, and deletions were corrected according to the mapping depth. Finally, we obtained a total of 249,226,675 contigs with an average N50 of 3,964 bp and an average length of 1,392 bp, totaling 337.5 × 10^9^ bp. Next, Prodigal [46] (v.2.6.3) was used to predict ORFs with the parameter “-p meta,” generating 469,651,662 ORFs with an average length of 621 bp, and 32.2% of the genes were identified as complete. ORFs less than 100 bp long obtained from the 370 samples were discarded, and the others were clustered using CD-HIT [47] (v.4.8.1, parameter: -n 9 -g 1 -c 0.95 -G 0 -M 0 -d 0 -aS 0.9), resulting in a nonredundant microbial gene catalog comprising 154,335,274 genes, referred to as the RGMGC (Additional file 2: Table S3). To assess the gene richness in the RGMGC, we generated species accumulation curves in each sample of 10 regions using the function “specaccum” in the R vegan package [48] (v.2.5-6), and coverage of the cumulative samples in each region was calculated as *C* = 1 – *n*/*N*, where *C* is the coverage, *n* is the number of genes that have been sampled once, and *N* is the total number of genes in the sample.

### Comparison of RGMGC with public datasets

To compare the microbial gene catalogs among ruminants, we also constructed nonredundant microbial gene catalogs for each ruminant species using CD-HIT [47] (v.4.8.1, parameter: -n 9 -g 1 -c 0.95 -G 0 -M 0 -d 0 -aS 0.9) and identified 35,513,583, 34,391,074, 32,856,686, 20,976,610, 24,217,232, 13,689,201 and 7,661,619 genes for dairy cattle, water buffalo, yak, goat, sheep, roe deer, and water deer, respectively (Additional file 2: Table S3). Then, we constructed nonredundant gene catalogs of Bovinae (97,393,650), Caprinae (40,885,116) and Cervidae (19,560,218). For the nonredundant microbial gene catalog of each GIT region, the assembled contigs from the same GIT regions of seven ruminant species were clustered using CD-HIT [47] (v.4.8.1, parameter: -n 9 -g 1 -c 0.95 -G 0 -M 0 -d 0 -aS 0.9) (Additional file 2: Table S3). To assess the representativeness of the ruminant GIT gene catalog in this study, we first compared the RGMGC with two rumen metagenomic datasets previously published by Hess et al. (2.5 M genes) [15] and Li et al. (13.8 M genes) [13] based on protein sequence identity for reduced variability using CD-HIT [47] (v.4.8.1, parameter: -n 5 -c 0.95 -G 0 -g 1 -M 0 -d 0 -aS 0.9). Next, we compared the RGMGC to a recently published large protein database (9.45 M protein clusters at a similarity cutoff of 100%) for the rumen MAG dataset from Stewart et al. [12]. We also compared the RGMGC with monogastric animals, including humans (9.9 M genes) [22], pigs (7.7 M genes) [23], and chickens (9.04 M genes) [24]. The catalogs for 10 GIT regions were compared based on protein sequence identity using CD-HIT [47] (v.4.8.1, parameter: -n 5 -c 0.95 -G 0 -g 1 -M 0 -d 0 -aS 0.9).

### Taxonomic classification and functional annotation

Entries in all the gene catalogs were subjected to taxonomic and functional assignment using DIAMOND [49] (v.0.9.22) based on BLASTP searches against the NCBI-NR (October 2018; approximately 550 M sequences), eggNOG [50] (v.4.5.1) and KEGG [51] (v.90.0) databases (parameter: --evalue 0.00001 --max-target-seqs 10). Each putatively encoded protein was assigned to an orthologous group by the highest scoring annotated hit. CAZymes were annotated by using HMMER [52] (v.3.2.1) to match protein sequences to entries in the hidden Markov model (HMM) libraries of CAZyme families downloaded from the CAZy database [53] (v.7; http://www.cazy.org/). The high-quality reads from each sample were aligned against the gene catalogs using BWA-MEM [40] (v.0.7.17), and abundance profiles of genes (alignment length ≥ 50 bp and sequence identity > 95%) were calculated in transcripts per million (TPM) [54], with corrections for variations in gene length and mapped reads per sample. TPM is calculated as

$$ TPM=\frac{N_g}{L_g}\times \frac{1}{\sum j\ \frac{N_j}{L_j}}\times {10}^6 $$

where *N*_*g*_ is the read count, i.e., the average number of reads mapped to the *g* gene; and *L*_*g*_ is the gene length, i.e., the number of nucleotides in the *g* gene. The index *j* stands for the set of all genes determined in a catalog, and *g* is an index indicating a particular gene [54]. The relative abundances of taxa, COGs, KOs, and CAZymes were calculated from the abundances of annotated genes [22]. Briefly, for the taxonomic (phylum and genus) profiles, we used phylogenetic assignment of each annotated gene from the RGMGC and summed the relative abundances of genes from the same phylum or genus to produce the abundance of each phylum or genus. The profile of each COG, KO, and CAZyme was calculated using the same process. The relative abundance of a COG category, KEGG pathway, and CAZyme family was calculated from the summation of the relative abundances of its contained COGs, KOs, and CAZymes, respectively.

### Metagenomic binning

To gain deep insights into the ruminant GIT microbiome, we took a metagenomic binning approach. To recover more assembled contigs, except for the individual assembly of each sample, we also co-assembled the high-quality reads from the same GIT regional samples in each ruminant species using MEGAHIT [41] (v.1.1.1, parameter: --min-contig-len 500 -t 40). The contigs from both single-sample assemblies and the co-assemblies (> 1.5 kb) were used for metagenomic binning independently, based on the sequence configurations and coverage depth using three methods with default parameters: MaxBin [55] (v.2.2.4), MetaBAT2 [56] (v.2.11.1), and CONCOCT [57] (v.0.4.0). DAS Tool [58] (v.1.1.1) was then applied to integrate the MAGs generated from the different methods. Single-sample binning produced a total of 87,410 bins, and co-assembly binning produced an additional 28,728. The completeness and contamination of all 116,138 bins were estimated using CheckM [25] (v.1.0.7) based on the lineage_wf workflow, which generated 28,543 MAGs that met or exceeded the medium-quality thresholds (≥ 50% completeness and < 10% contamination), and 7698 were estimated to be near-complete (> 90% completeness and < 5% contamination) (Additional file 4: Table S13). Quality scores for each MAG were calculated in terms of the level of completeness − 5 × the contamination according to a previous study [26]. Then, the 28,543 MAGs were dereplicated with a 99% ANI cutoff using dRep [59] (v.2.5.4; parameter: -p 72 --ignoreGenomeQuality -pa 0.95 -sa 0.99 -cm larger), and 10,373 nonredundant MAGs were obtained. Additional statistics for each nonredundant genome are listed in Additional file 4: Table S16, including the contig N50, number of contigs, average contig length, number of ORFs, numbers of tRNA and rRNA genes, and contig read depth. ORFs were predicted using Prodigal [46] (v.2.6.3) with the parameter “-p single.” tRNAs were identified using tRNAscan-SE [60] (v.2.0.4) and rRNA genes using Barrnap (v.0.9-dev; https://github.com/tseemann/barrnap) with options “–reject 0.01 –evalue 1e-3.” Genome size was corrected for completeness and contamination according to the previously reported equation *Ĝ* = *G* ∗ 100/*C* − (*G* ∗ *T*/100), where *Ĝ* is the estimated genome size of a MAG, *G* is the observed genome size, *C* is the estimated percent completeness, and *T* is the estimated percent contamination [61]. We estimated the contig read-depth of 10,373 MAGs in each sample using metaWRAP [62] (v.1.3) with a “quant_bins” module. The contigs from all bins were first collected as a reference, and reads from each sample were aligned to the assembly. The average abundance of each MAG in each sample was calculated according to the TPM calculation process (as mentioned before), taking the length-weighted average of the contig abundances in each MAG (Additional file 4: Table S16).

### Detecting non-prokaryotic bins

Although this study mainly focused on the bacterial and archaeal diversity in the recovered MAGs, we further investigated how many of our bins represented known eukaryote or viral sequences that form part of the ruminant GIT microbiota. As CheckM is unable to evaluate nonprokaryotic genomes, we first compared all bins not assigned to either bacteria or archaea (*n* = 13,979) against the GenBank collection of all fungal (*n* = 2,647) and protozoan (*n* = 468) genomes (Additional file 4: Table S19). FastANI [63] (v.1.2) was used to calculate the ANI, and MUMmer [64] (v.3.0) was used to determine the fraction of the MAG aligned to reference genomes. We detected that eight bins had at least 50% of their genome aligned to known protozoan organisms. As viral sequences were rarely binned together and instead were binned with prokaryotic or eukaryotic sequences, we screened the original metagenome assemblies for the presence of viral contigs. Using VirFinder [65] (v.1.1), we detected 310,661 viral contigs ≥ 5 kb in length (score ≥ 0.9 and *P* < 0.05).

### Species-level clustering of reference genomes and MAGs

We downloaded 41,369 bacterial and 2163 archaeal representative genomes in the GenBank database on August 25, 2019 (Additional file 4: Table S19) from a wide range of environmental or gut studies and 7,052 previously published MAGs and genomes isolated from the ruminant GIT (referred to as the RMG; Additional file 4: Table S20). All these genomes were used as reference genomic datasets for the identification of novel microbial genomes from the 10,373 MAGs using FastANI [63] (v.1.2). Based on a species-level criterion [29] of ≥ 60% alignment of the sequence fraction with ≥ 95% ANI, 8,745 MAGs did not match any available reference genomes, including 1886 near-complete genomes and 6859 of medium-quality (quality scores: 4065 > 50 and 2794 ≤ 50), and were thus referred to as the USGs. We also retrieved 635 ruminant metagenomes from samples analyzed in 16 studies (~ 11.3 Tb in total; Additional file 2: Table S4) published by August 2019. All the sequenced reads from retrieved metagenomic data and our study were mapped to three datasets (GenBank, RMGs, and our USGs) using BWA-MEM [40] (v.0.7.17) to assess the taxonomic classification after filtering out contaminating DNA and quality control. Four combined databases were used to determine the read classification rates: a common database consisting of the bacterial and archaeal genomes in GenBank, the GenBank database plus the RMGs, the GenBank database plus the USGs, and all three datasets.

### Phylogenetic, taxonomic, and functional analyses of MAGs

To determine the phylogenetic affiliation and diversity of the 10,373 MAGs, we computed the total branch length based on entries in published ruminant genomes. First, we collected 6421 rumen microbial genomes from previous studies [12, 13, 15, 26, 66, 67] and 631 genomes of cultured isolates from the Hungate1000 project [10] and other studies by October 2019 (Additional file 4: Table S20). Next, ORFs were predicted in 7052 public genomes using Prodigal [46] (v.2.6.3; parameter: -p single). PhyloPhlAn [30] (v.1.0) was applied to build a phylogenetic tree of the total of 17,425 microbial genomes by aligning the individual proteins from the protein sets recovered from the input genomes using MUSCLE [68] (v.3.8.31). Then, the most discriminative positions in each protein alignment were concatenated into a single long sequence to reconstruct a maximum-likelihood tree using FastTree [69] (v.2.1.9). The phylogenetic trees were visually inspected using Evolview [70] (v.3) and iTol [71] (v.4.3.1). The final tree was used to estimate the total branch length (phylogenetic diversity, PD) and increased total branch length (phylogenetic gain, PG) for the USG collection using GenomeTreeTk [26] (v.0.0.54):

$$ \mathrm{PG}=1-\frac{\mathrm{PD}\ \left(\mathrm{tree}\ \mathrm{subset}\ \mathrm{excluding}\ \mathrm{USG}\right)}{\mathrm{PD}\ \left(\mathrm{complete}\ \mathrm{tree}\right)} $$

All genomes were taxonomically annotated using GTDB-Tk [27] (v.0.1.6) based on the Genome Taxonomy Database (http://gtdb.ecogenomic.org/), which produced standardized taxonomic labels that were used for the analysis in this study [61].

The 10,373 MAGs and 7052 published genomes were functionally analyzed as follows. All the predicted genes were functionally characterized using Genome-properties [72] (v.2.0), an integrated annotation system utilizing the InterPro (v.5.30-69.0) database to assign functional attributes to each genome. The GPs of each genome classified as “Complete,” “Partial,” and “Absent” were converted to numeric values (2, 1, and 0, respectively), and those that significantly differed between different groups of genomes were analyzed with a two-tailed Chi-squared test [29]. We also matched the protein sequences encoded by each genome using HMMER [52] (v.3.2.1) to the HMM libraries of CAZyme families and then followed the PULpy [73] (v.1.0) pipeline for PUL predictions. The presence of microbial secondary metabolite BGCs in each genome was predicted using antiSMASH [74] (v.5.1.2; parameter: –knowclusterblast), and the novel clusters of BGCs were determined as BGCs without a positive match in the Minimum Information about a Biosynthetic Gene cluster repository.

### Co-occurrence network

To construct a co-occurrence network of the 10,373 MAGs in the ruminant GIT, we first calculated the correlations between two MAGs based on their abundances in all samples with the R package Hmisc [75] (v.4.4.0) using the Spearman correlation test. A Spearman’s rho with asymptotic measure-specific *P* value was generated to assess the associations between MAGs and was then supported by the assessment of significance with a |Spearman’s rank correlation coefficient| > 0.85 and *P* < 0.01 [76]. Co-occurrence network modules were then inferred by using the weighted correlation network analysis with the R package WGCNA [77] (v.1.69), and networks were graphed using Gephi [78] (v.0.9.2) based on the Fruchterman-Reingold algorithm. The network was divided into different subnetworks according to the retrieved region of each MAG. Hub genomes in the network were defined as those at nodes with the highest connectivity in each network according to the eigenvector centrality for each genome performed using Gephi.

### Ordination analysis

PCoA was performed to reveal the differences between pairs of samples or MAGs based on their taxonomic and functional profiles using the Bray-Curtis dissimilarity matrix, and then the differences between groups were assessed using the ANOSIM test in the R package vegan [48] (v.2.5-6) with 9,999 permutations. We used variance partitioning analysis by the “varpart” program in the R vegan package to assess the variances in distances among samples explained by GIT regions and ruminant species. The taxonomic (genus) and functional (COGs, KOs and CAZymes) modules present in 90% of the individuals in each gene catalog were used in comparisons [23] and visualized using UpSetR [79] (v.1.4.0) with Venn diagrams. Alpha (Shannon index) and beta (Bray-Curtis dissimilarity) diversities were compared using the R vegan package. All trilinear plots were built in the R environment using the ggtern and ggplot2 packages.

### Methanogenic archaea and methanogenesis

A maximum-likelihood tree of the 318 archaeal genomes including 160 MAGs from this study and 158 previously published genomes was constructed using PhyloPhlAn [30] (v.1.0) with the parameter “-mode denovo.” Protein sequences encoded by our 10,373 MAGs were also screened against the KEGG (v.90.0) database and HydDB [80] to identify catalytic subunits of the three classes of hydrogenases (NiFe-, FeFe-, and Fe-hydrogenases) by BLASTP with an e-value threshold of 1e-50, coverage values exceeding 90% and identity values exceeding 50% [18]. Genes encoding subunits of terminal reductases or other metabolic enzymes (including *acsB*, *aprA*, *asrA*, *cooS*, *dmsA*, *dsrA*, *frdA*, *hydB*, *mcrA*, *napA*, *narG*, *nifH*, *nirK*, *nirS*, *nosZ*, *nrfA*, and *sdhA*) were identified by BLASTP searches against respective gene sequences (with an e-value threshold of 1e-50, coverage values exceeding 90% and identity values exceeding 50 or 60%). In addition, *ccoN*, *coxA*, *cydA*, *cyoA*, *codH*, *nirB*, *fdhA*, and *norB* genes were identified by HMMER [52] (v.3.2.1) searches against Pfam (v.31.0) and TIGRFAMs (v.15.0) databases (with an e-value threshold of 1e-10 and separate cutoff scores).

### Analysis of cattle feed efficiency data

We merged the RMG and USG datasets as a taxonomic database and then used it to assign previously published FE data from high- and low-FE beef cattle samples [35] to different taxonomic levels using BWA-MEM [40] (v.0.7.17). We detected significantly different taxa in the two sets of samples at the phylum, class, order, family, genus, and species levels using the test applied in the cited study. For genomic differential analysis, we used the resulting read count data as input for modeling with the DESeq2 [81] (v.1.28.1). We found 1338 differentially enriched genomes between high- and low-FE cattle based on a log_2_^fold-change^ > 1 and *P* < 0.05, and then selected 410 near-complete genomes for the following genomic comparison. Metagenome-wide association studies were used to estimate correlations between genomes and FE, with |Pearson correlation coefficients| > 0.5 and *P* < 0.05. Among these 410 genomes, we detected 35 positively and 2 negatively correlated with FE, respectively. Finally, we compared the differentially enriched genomes in terms of CAZyme-encoding genes. Detailed information on these 410 genomes is presented in Additional file 7: Table S31.

## Additional files

**Additional file 1: Fig. S1.** Computational pipeline for gene catalog and assembly of MAGs. **Fig. S2.** DNA contamination statistics. **Fig. S3.** Assembly statistics. **Fig. S4.** Comparison of the RGMGC to the public datasets. **Fig. S5.** Coverage of the RGMGC. **Fig. S6.** Comparison of the dominant microbial taxa at the genus level among GIT regions. **Fig. S7.** Functional structure of the GIT microbiome. **Fig. S8.** Gene diversity in microbial communities across the ruminant GIT. **Fig. S9.** CheckM quality assessment. **Fig. S10.** Distribution of 10,373 genomes across the ruminant GIT. **Fig. S11.** Variations in enrichment of MAGs among GIT regions. **Fig. S12.** Comparative analysis of genomes of the CAG-110 genus. **Fig. S13.** Species-level clustering of reference genomes and MAGs. **Fig. S14.** Distribution of the 8,745 USGs across the ruminant GIT. **Fig. S15.** Taxonomic composition of the 8,745 USGs. **Fig. S16.** Biosynthetic gene clusters found in the human gut species. **Fig. S17.** Differences in GP profiles between the USGs and RCGs. **Fig. S18.** Comparison of the USGs and RCGs in the prevalent phyla. **Fig. S19.** Phylogenetic tree of the 194 proteobacteria genomes. **Fig. S20.** Phylogenetic tree of mutualistic archaea. **Fig. S21.** Distributions of hydrogenases and associated terminal reductases in the 10,373 MAGs. **Fig. S22.** Associations of GIT microbial species with cattle feed efficiency (FE).

**Additional file 2: Table S1.** Background information on the 370 ruminant GIT content samples. **Table S2.** Assembly results of the 370 samples. **Table S3.** Description of the nonredundant ruminant GIT microbial gene catalogs. **Table S4.** Published ruminant metagenomic datasets used in this study. **Table S5.** The 635 published ruminant metagenomic samples mapped to the RGMGC. **Table S6.** Taxonomic composition of the RGMGC based on the NCBI-NR annotation at the phylum and genus levels. **Table S7.** Comparison of the microbial KEGG pathways between ruminant and monogastric animals. **Table S8.** Comparison of the relative abundance of the microbial taxa among the GIT regions.

**Additional file 3: Table S9.** Differences in functional categories among the GIT regions.

**Additional file 4: Table S10.** Comparison of the levels of COG functional modules of the microbiome across the ruminant GIT regions. **Table S11.** Comparison of the levels of KO functional modules of the microbiome across the ruminant GIT regions. **Table S12.** Comparison of the levels of CAZyme functional modules of the microbiome across the ruminant GIT regions. **Table S13.** Quality information and assessment of 28,543 MAGs produced in this study. **Table S14.** Bins that aligned to fungal or protozoan genomes from GenBank. **Table S15.** Quality information for 310,661 viral contigs generated from our dataset. **Table S16.** Genomic statistics for 10,373 nonredundant MAGs from our dataset. **Table S17.** Taxonomic classification and genome properties of 367 MAGs aggregated in Module 47 of Fig. 4. **Table S18.** Taxonomic information for the top 10 Hub genomes in each GIT region. **Table S19.** The 41,369 bacterial, 2,163 archaeal, 2,647 fungal and 468 protozoan reference genomes from GenBank used in this study. **Table S20.** Collection of 7,052 previously published ruminant microbial genomes. **Table S21.** Quality assessment and taxonomic classification of 8,745 uncultured candidate bacterial and archaeal species. **Table S22.** Read classification rate of 635 published metagenomic samples and 370 GIT samples in this study using GenBank, RMG and USG. **Table S23.** The 850,749 CAZyme-predicted proteins from the 8,745 USGs listed in Additional file 4: Table S18. **Table S24.** The 12,578 predicted PULs and taxonomic classification of 1,772 USGs. **Table S25.** Number and characteristics of biosynthetic gene clusters identified in the USGs. **Table S26.** Comparison of GPs in the genomes assigned to the phylum Proteobacteria. **Table S27.** Taxonomic classification and distribution of 160 archaeal MAGs among the GIT regions. **Table S28.** Taxonomic classification and counts of predicted hydrogenases among the 6,152 MAGs.

**Additional file 5: Table S29.** Taxonomic classification and counts of predicted hydrogenases and enzymes associated with methanogenesis, acetogenesis, fumarate reduction, sulfidogenesis and nitrate ammonification.

**Additional file 6: Table S30.** Significant differences in taxa between low- and high-FE cattle based on the RMG and USG datasets as a taxonomic database.

**Additional file 7: Table S31.** Taxonomic classification and counts of CAZyme genes in the 410 differentially enriched genomes in high- and low-FE cattle.

## Data Availability

Raw sequence reads and metagenome-assembled genomes for all samples are available under European Nucleotide Archive (ENA) project PRJNA657455 and PRJNA657473. The protein and ORF sequences of all MAGs and USGs have been deposited in Figshare (DOI: 10.6084/m9.figshare.14176574). All the gene catalogs, annotation information, abundance profiles, assemblies, and predicted ORFs from this study are available at http://www.rummeta.com. The workflow and scripts used to generate the gene catalogs and functional annotations are available at https://github.com/orctyr/RGMGC. Genomic analysis of the MAGs and USGs is described in a pipeline at https://github.com/orctyr/MAGs-pipeline.
