# Correction: An integrated gene catalog and over 10,000 metagenome-assembled genomes from the gastrointestinal microbiome of ruminants

**DOI:** 10.1186/s40168-022-01426-5

**Published:** 2022-12-15

**Authors:** Fei Xie, Wei Jin, Huazhe Si, Yuan Yuan, Ye Tao, Junhua Liu, Xiaoxu Wang, Chengjian Yang, Qiushuang Li, Xiaoting Yan, Limei Lin, Qian Jiang, Lei Zhang, Changzheng Guo, Chris Greening, Rasmus Heller, Le Luo Guan, Phillip B. Pope, Zhiliang Tan, Weiyun Zhu, Min Wang, Qiang Qiu, Zhipeng Li, Shengyong Mao

**Affiliations:** 1 Laboratory of Gastrointestinal Microbiology, College of Animal Science and Technology, Nanjing Agricultural University, Nanjing, China; 2 College of Animal Science and Technology, Jilin Agricultural University, Changchun, China; 3 School of Ecology and Environment, Northwestern Polytechnical University, Xi’an, China; 4 Shanghai BIOZERON Biotechnology Company Ltd, Shanghai, China; 5 Department of Special Economic Animal Nutrition and Feed Science, Institute of Special Animal and Plant Sciences, Chinese Academy of Agricultural Sciences, Changchun, China; 6 Buffalo Research Institute, Chinese Academy of Agricultural Sciences, Nanning, China; 7 CAS Key Laboratory for Agro-Ecological Processes in Subtropical Region, Institute of Subtropical Agriculture, Chinese Academy of Sciences, Changsha, China; 8 Biomedicine Discovery Institute, Department of Microbiology, Monash University, Clayton, Australia; 9 Section for Computational and RNA Biology, Department of Biology, University of Copenhagen, Copenhagen, Denmark; 10 Department of Agricultural, Food and Nutritional Science, University of Alberta, Edmonton, Canada; 11 Faculty of Biosciences, Norwegian University of Life Sciences, Aas, Norway

**Correction: Microbiome 9, 137 (2021)**

**https://doi.org/10.1186/s40168-021-01078-x**

In the original article [1], it has been reported that the URL "http://www.rummeta.com" found in the Availability of Data and materials section leads to an incorrect site. The new URL should be "http://rummeta.njau.edu.cn/."

The original article has been updated.
