# Supplementary material for: An integrated gene catalog and over 10,000 metagenome-assembled genomes from the gastrointestinal microbiome of ruminants
Source: Microbiome. 2021 Jun 12;9:137. doi: 10.1186/s40168-021-01078-x (PMC8199421; doi:10.1186/s40168-021-01078-x)
Supplement: Supplementary file 2 — Additional file 1: Fig. S1. Computational pipeline for gene catalog and assembly of MAGs. Fig. S2. DNA contamination statistics. Fig. S3. Assembly statistics. Fig. S4. Comparison of the RGMGC to the public datasets. Fig. S5. Coverage of the RGMGC. Fig. S6. Comparison of the dominant microbial taxa at the genus level among GIT regions. Fig. S7. Functional structure of the GIT microbiome. Fig. S8. Gene diversity in microbial communities across the ruminant GIT. Fig. S9. CheckM quality assessment. Fig. S10. Distribution of 10,373 genomes across the ruminant GIT. Fig. S11. Variations in enrichment of MAGs among GIT regions. Fig. S12. Comparative analysis of genomes of the CAG-110 genus. Fig. S13. Species-level clustering of reference genomes and MAGs. Fig. S14. Distribution of the 8,745 USGs across the ruminant GIT. Fig. S15. Taxonomic composition of the 8,745 USGs. Fig. S16. Biosynthetic gene clusters found in the human gut species. Fig. S17. Differences in GP profiles between the USGs and RCGs. Fig. S18. Comparison of the USGs and RCGs in the prevalent phyla. Fig. S19. Phylogenetic tree of the 194 proteobacteria genomes. Fig. S20. Phylogenetic tree of mutualistic archaea. Fig. S21. Distributions of hydrogenases and associated terminal reductases in the 10,373 MAGs. Fig. S22. Associations of GIT microbial species with cattle feed efficiency (FE). [file 40168_2021_1078_MOESM2_ESM.docx]

**
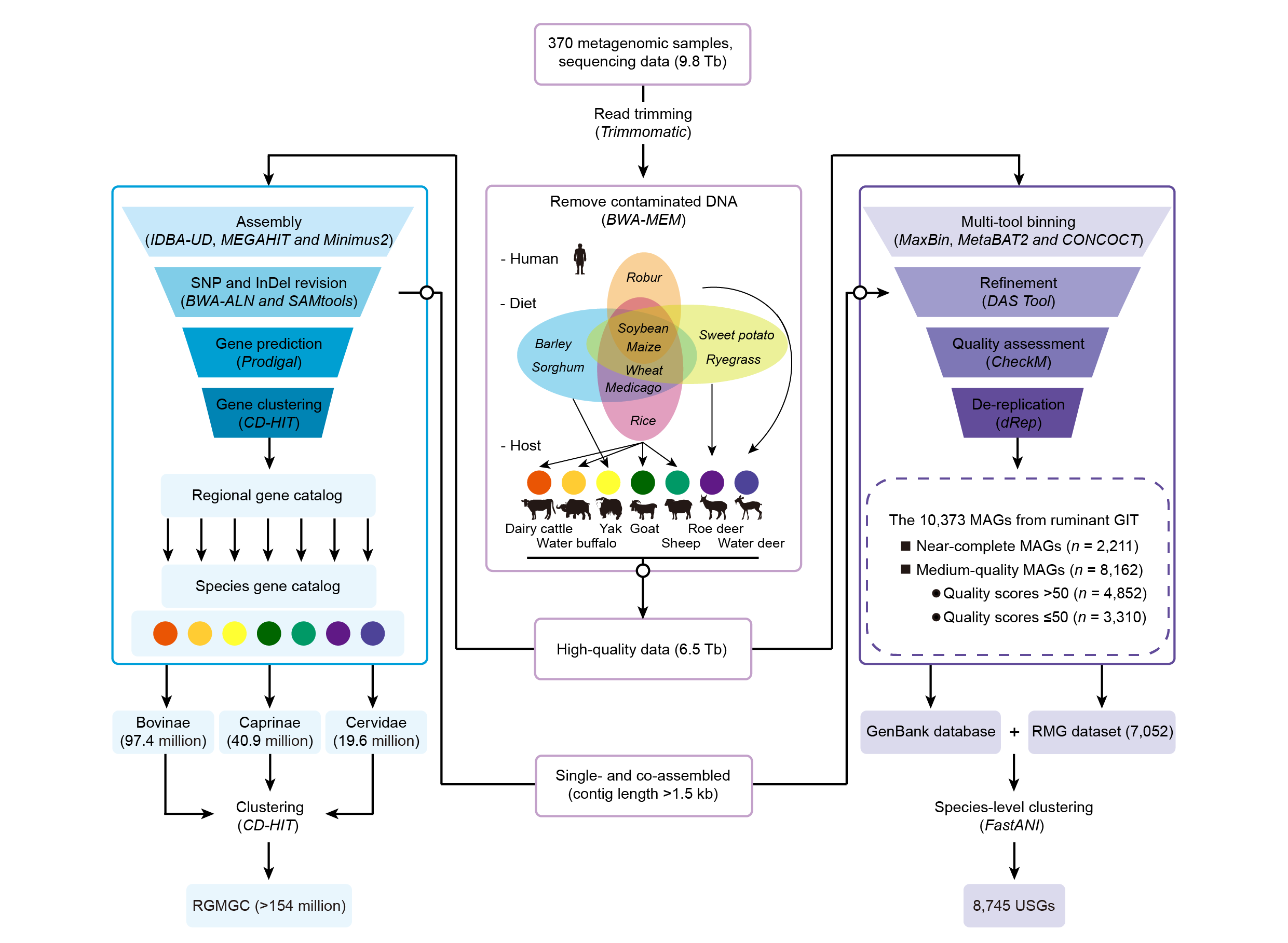
**

**Fig. S1** **Computational pipeline for gene catalog and assembly of MAGs.** Overview of the methodology used to filter metagenomic sequencing reads, construct the largest (>154 million genes) gene catalog of the ruminant GIT microbiome (RGMGC) and retrieve over 8,000 unknown species-level genomes (USGs) from metagenomes spanning the ruminant GIT. We used the genome sets of hosts, including dairy cattle (*Bos Taurus*, GCA_002263795.2), water buffalo (*Bubalus bubalis*, GCA_003121395.1), yak (*Bos mutus*, GCA_000298355.1), goat (*Capra hircus*, GCA_001704415.1), sheep (*Ovis aries*, GCA_002742125.1), roe deer (*Capreolus pygargus*, GCA_000751575.1) and water deer (*Hydropotes inermis*, GCA_006459105.1); plants, including sorghum (*Sorghum bicolor*, GCA_000003195.3), wheat (*Triticum aestivum*, GCA_002220415.3), robur (*Quercus robur*, GCA_900291515.1), sweet potato (*Ipomoea batatas*, GCA_002525835.2), medicago (*Medicago truncatula*, GCA_000219495.2), rice (*Oryza sativa*, GCF_000005425.2), barley (*Hordeum vulgare*, GCA_900075435.2), maize (*Zea mays*, GCA_003185045.1 and GCA_000005005.6), soybean (*Glycine max*, GCA_000004515.4), and ryegrass (*Lolium perenne*, GCA_001735685.1); and human (*Homo sapiens*, GCA_000001405.28), as references for mapping to decrease the potential DNA contamination.


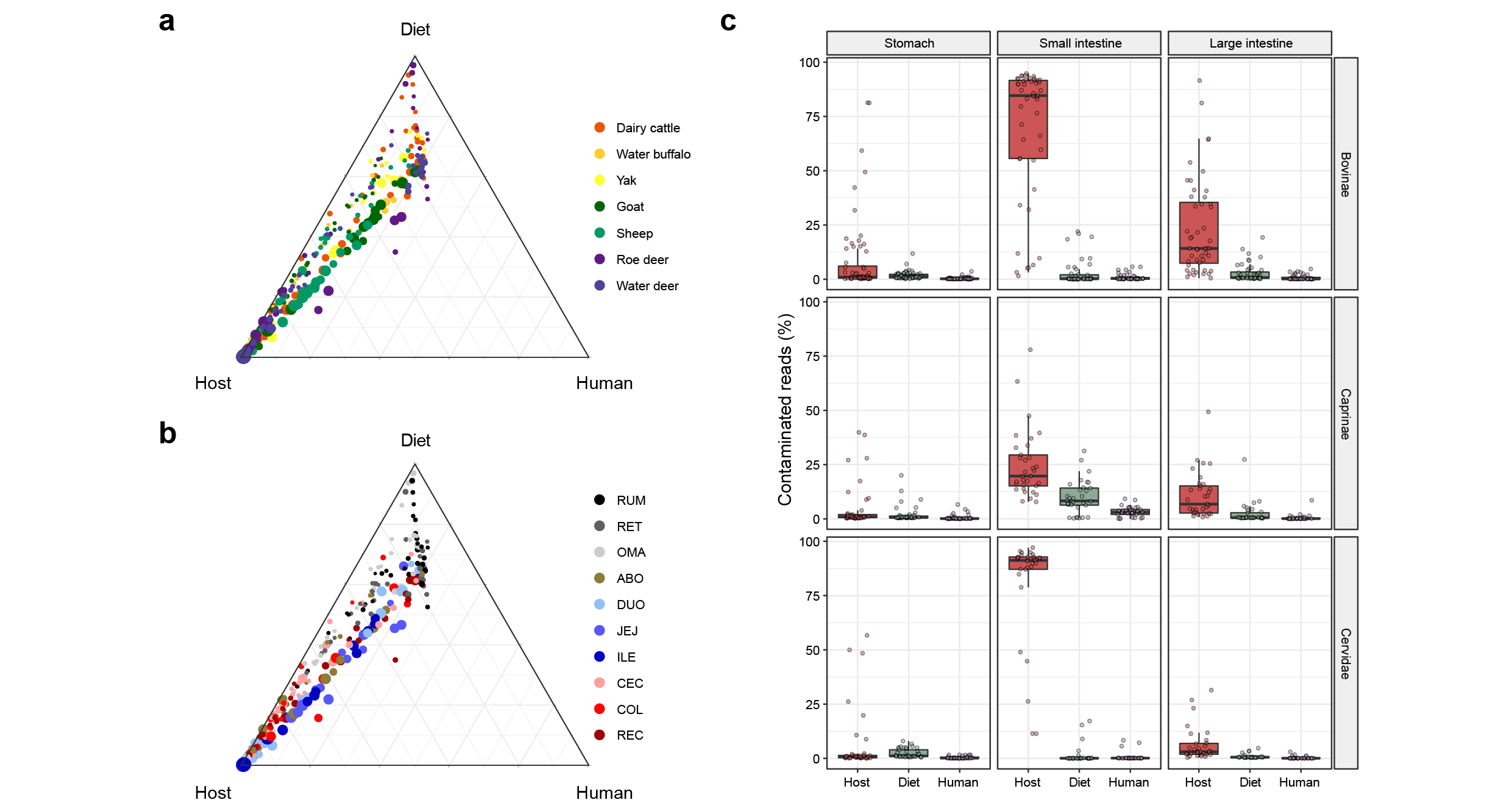


**Fig. S2 DNA contamination statistics.** **a** and **b** Ternary diagrams of host, human, and diet DNA contamination rates in color-coded samples from indicated species and GIT regions. Circle sizes indicate total DNA contamination levels. **c** Boxplots showing variations in these contamination rates among ruminant families (Bovinae, Caprinae and Cervidae) and GIT regions (stomach, small intestine and large intestine). In each box plot, the horizontal line indicates the median, and the whiskers indicate the lowest and highest points within 1.5× the interquartile ranges into the lower and upper quartiles, respectively. RUM, rumen; RET, reticulum; OMA, omasum; ABO, abomasum; DUO, duodenum; JEJ, jejunum; ILE, ileum; CEC, cecum; COL, colon; REC, rectum.


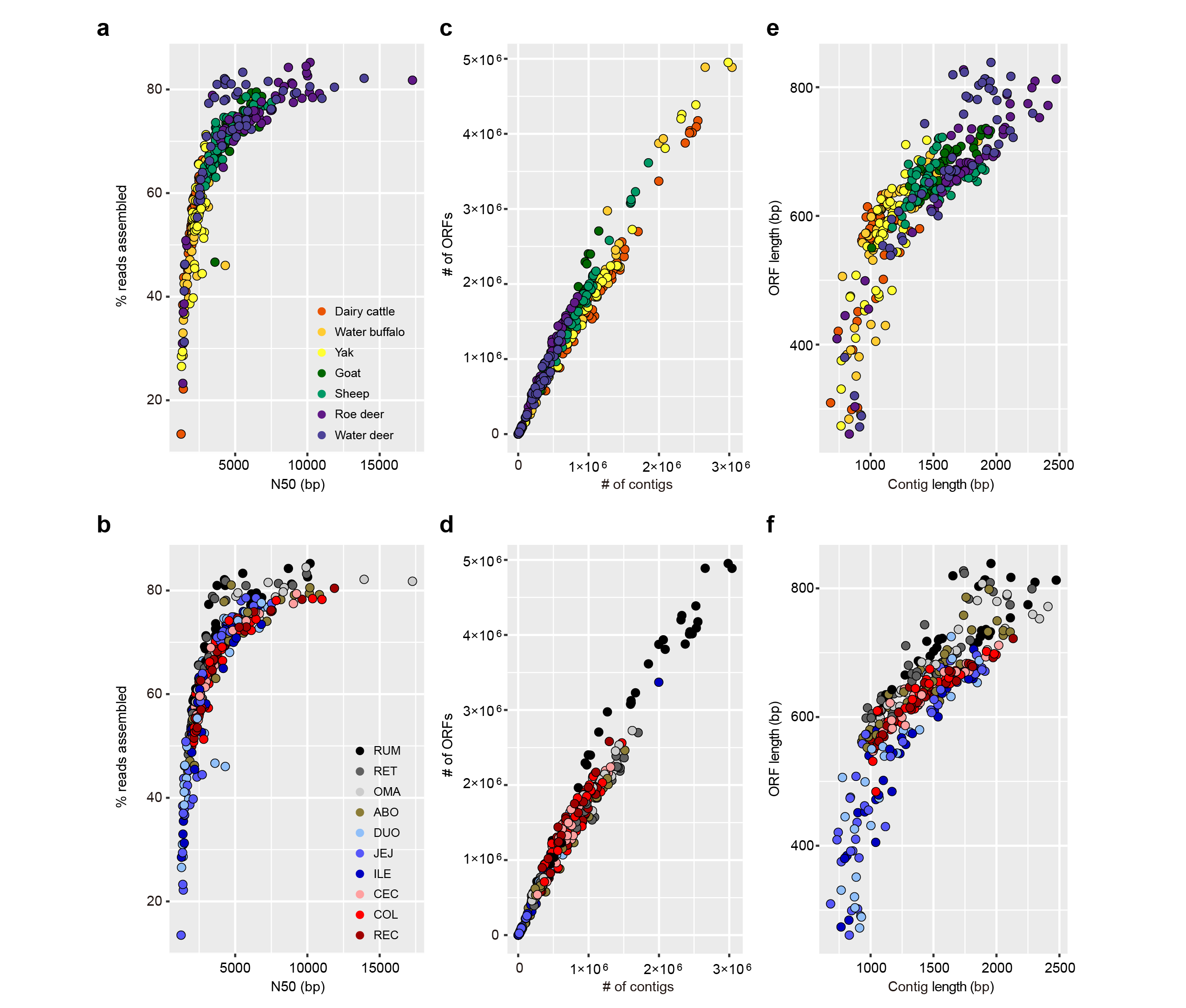


**Fig. S3 Assembly statistics.** Scatter diagrams showing percentages of assembled reads vs. N50 (**a** and **b**), number of ORFs vs. number of contigs (**c** and **d**), and average ORF length vs. average contig length (**e** and **f**) in color-coded samples from indicated species and GIT regions. ORFs, open reading frames. RUM, rumen; RET, reticulum; OMA, omasum; ABO, abomasum; DUO, duodenum; JEJ, jejunum; ILE, ileum; CEC, cecum; COL, colon; REC, rectum.


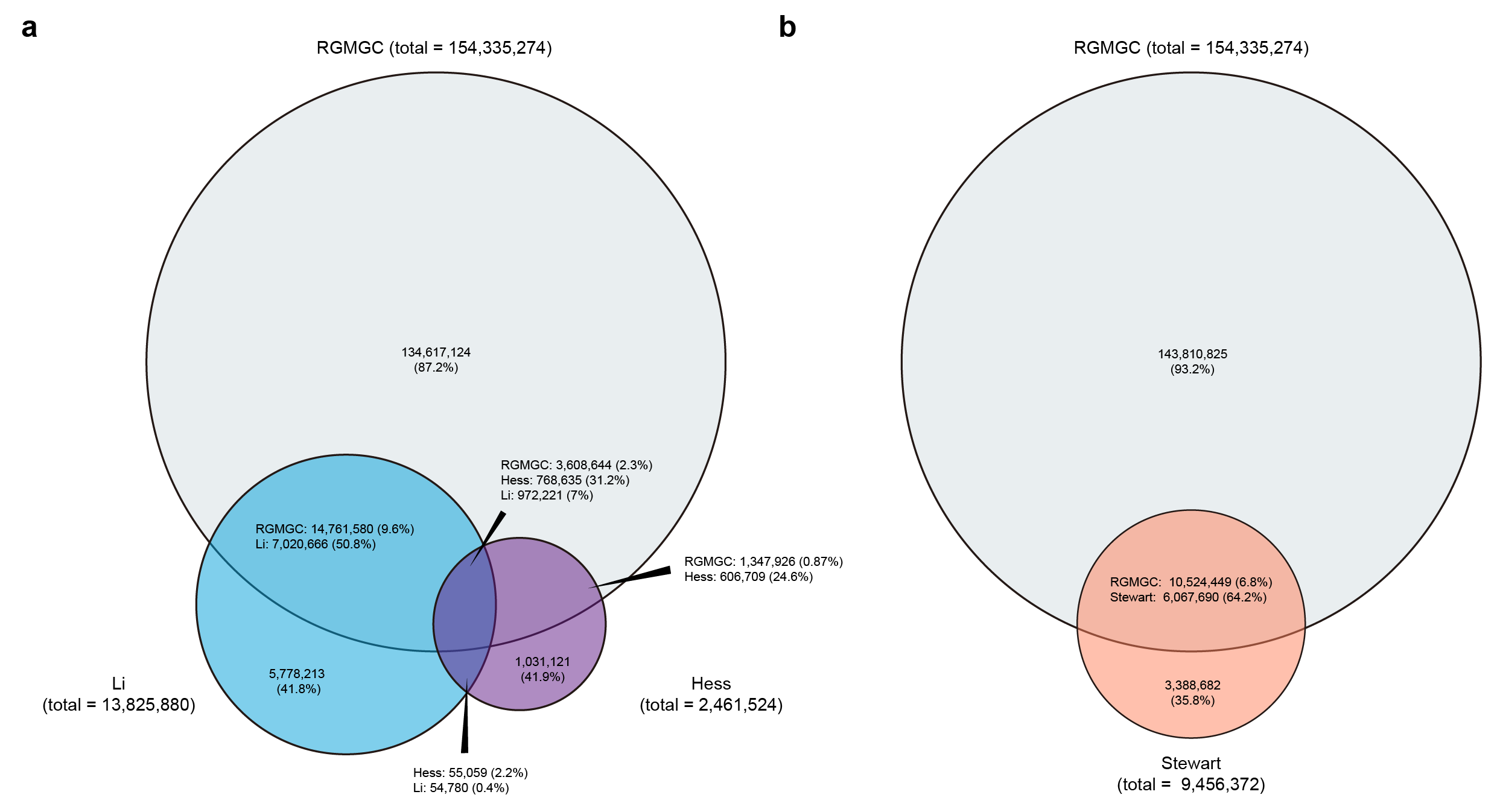


**Fig. S4 Comparison of the RGMGC to the public datasets.** **a** Venn diagram of genes shared by the RGMGC and previously published rumen microbial gene catalogs based on encoded protein sequence identity. Hess refers to [1], and Li refers to [2]. **b** Comparison of the RGMGC to recently published protein database for rumen MAG dataset from Stewart et al [3].

**
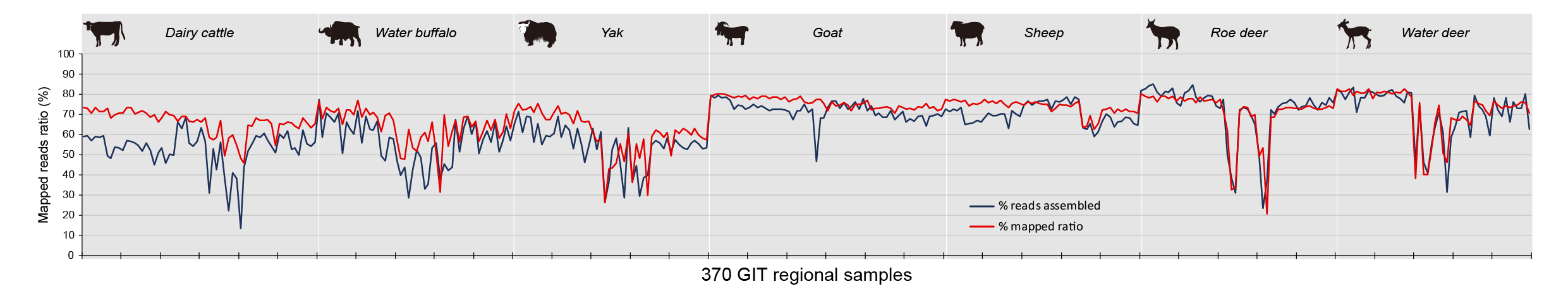
**

**Fig. S5 Coverage of the RGMGC.** The blue line shows the percentage of reads used to assemble in each sample. The red line shows the percentage of high-quality reads in 370 samples of the seven ruminant species that were mapped to the RGMGC.


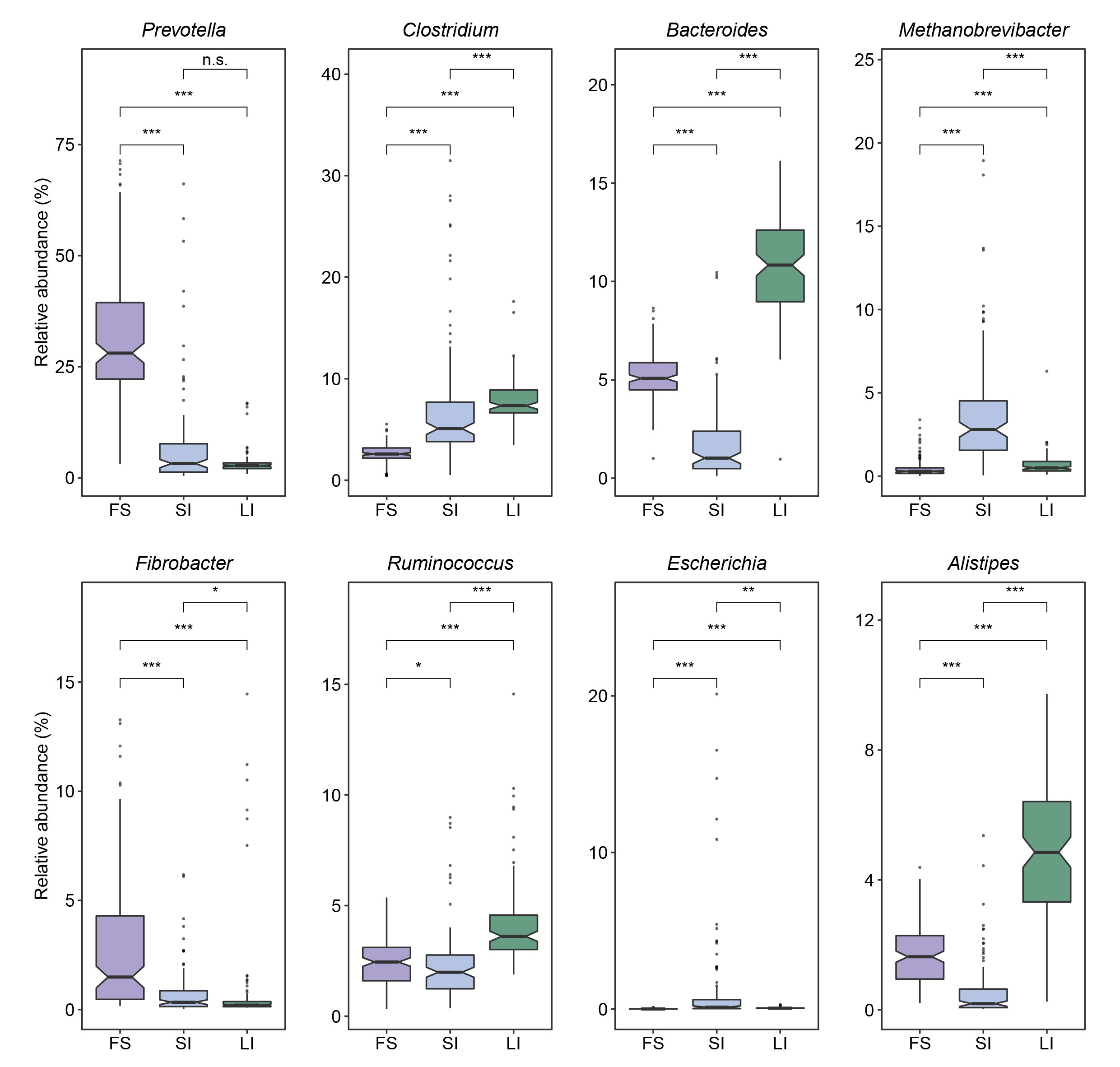


**Fig. S6 Comparison of the dominant microbial taxa at the genus level among GIT regions.** Boxplots showing the average abundances of indicated genera in microbial communities of the stomach (FS: rumen, reticulum, omasum and abomasum), small intestine (SI: duodenum, jejunum and ileum) and large intestine (LI: cecum, colon and rectum). Significant between-region differences based on the relative abundance of each cohort according to the Wilcoxon rank-sum test are indicated by asterisks (**P* < 0.05, ***P* < 0.01, ****P* < 0.001. n.s., not significant). In each box plot, the horizontal line indicates the median, and the whiskers indicate the lowest and highest points within 1.5× the interquartile ranges into the lower and upper quartiles, respectively.


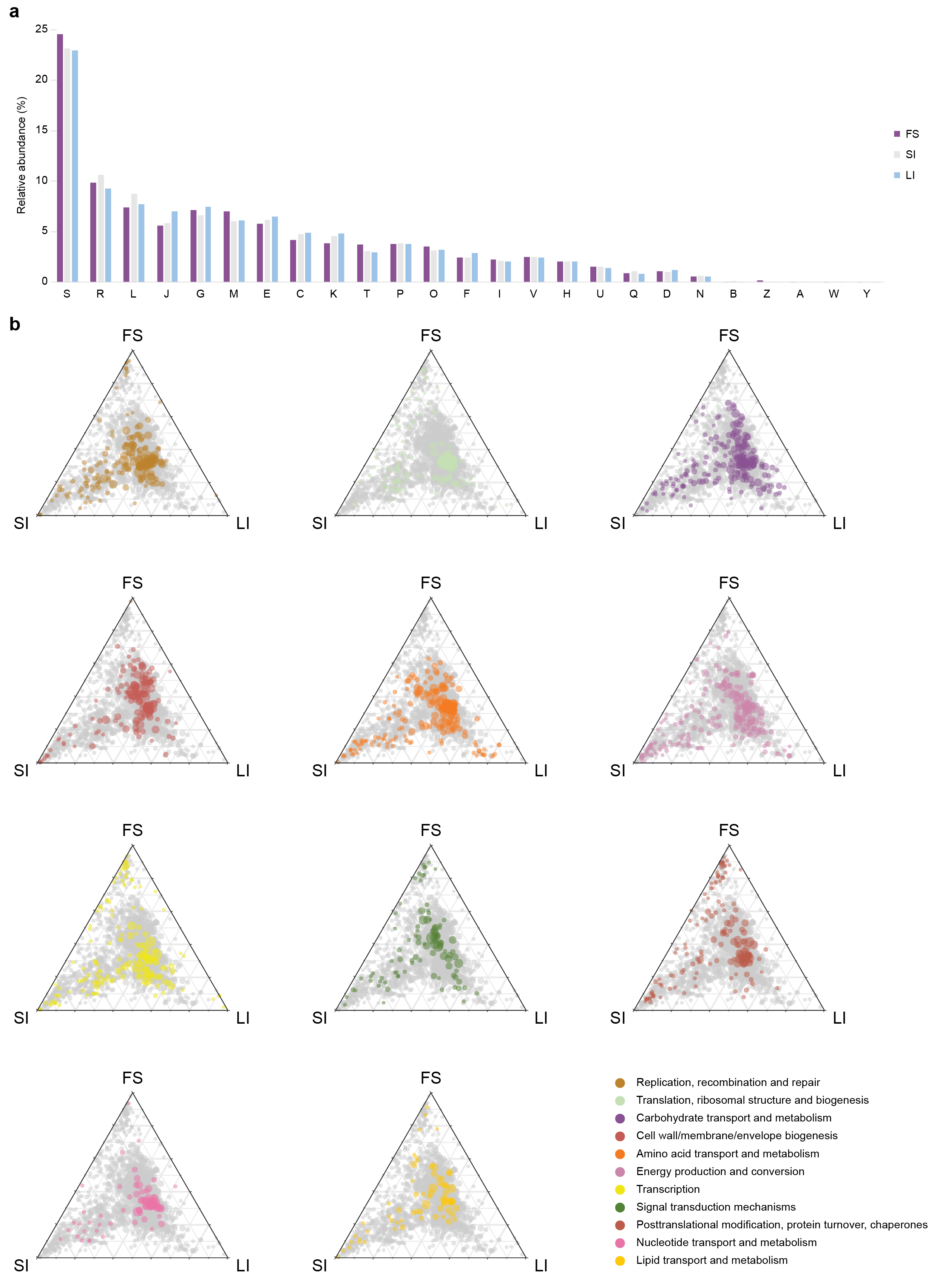


**Fig. S7 Functional structure of the GIT microbiome. a** Average abundances of functional components based on COG categories and **b** differences in enrichment of COG-assigned proteins between the stomach (FS: rumen, reticulum, omasum and abomasum), small intestine (SI: duodenum, jejunum and ileum) and large intestine (LI: cecum, colon and rectum). A, RNA processing and modification; B, chromatin structure and dynamics; C, energy production and conversion; D, cell cycle control, cell division, chromosome partitioning; E, amino acid transport and metabolism; F, nucleotide transport and metabolism; G, carbohydrate transport and metabolism; H, coenzyme transport and metabolism; I, lipid transport and metabolism; J, translation, ribosomal structure and biogenesis; K, transcription; L, replication, recombination and repair; M, cell wall/membrane/envelope biogenesis; N, cell motility; O, posttranslational modification, protein turnover, chaperones; P, inorganic ion transport and metabolism; Q, secondary metabolites biosynthesis, transport and catabolism; R, general function prediction only; S, function unknown; T, signal transduction mechanisms; U, intracellular trafficking, secretion, and vesicular transport; V, defense mechanisms; W, extracellular structures; Y, nuclear structure; Z, cytoskeleton.


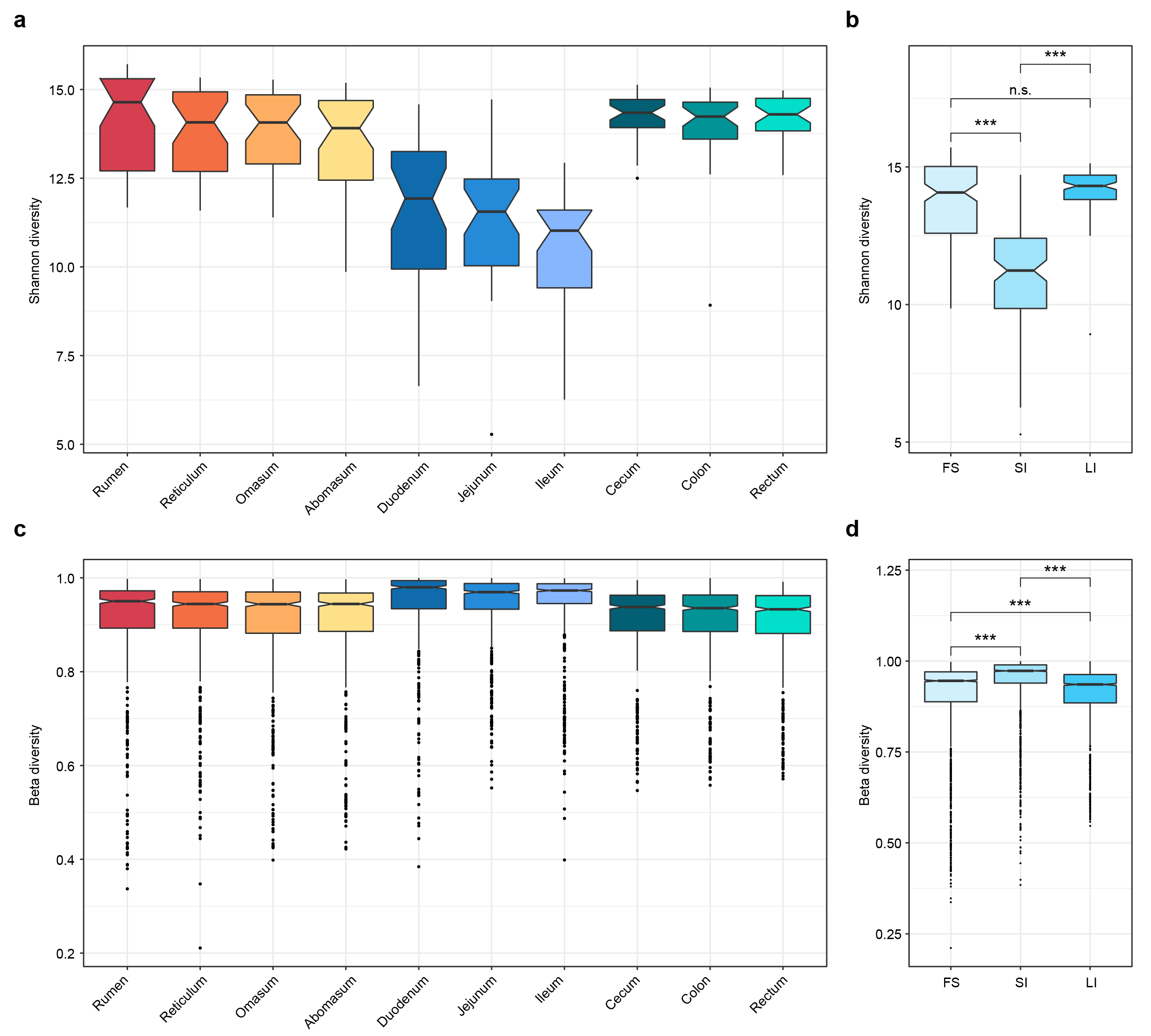


**Fig. S8 Gene diversity in microbial communities across the ruminant GIT.** Alpha diversity (Shannon index) (**a** and **b**) and beta diversity (Bray-Curtis) (**c** and **d**) based on nonredundant gene counts. Data are shown as box plots, where the horizontal line indicates the median and the whiskers indicate the lowest and highest points within 1.5× the interquartile ranges into the lower and upper quartiles, respectively. FS, stomach; SI, small intestine; LI, large intestine. Asterisks indicate significant differences between cohorts (**P* < 0.05, ***P* < 0.01, ****P* < 0.001. n.s., not significant) according to the Wilcoxon rank-sum test.


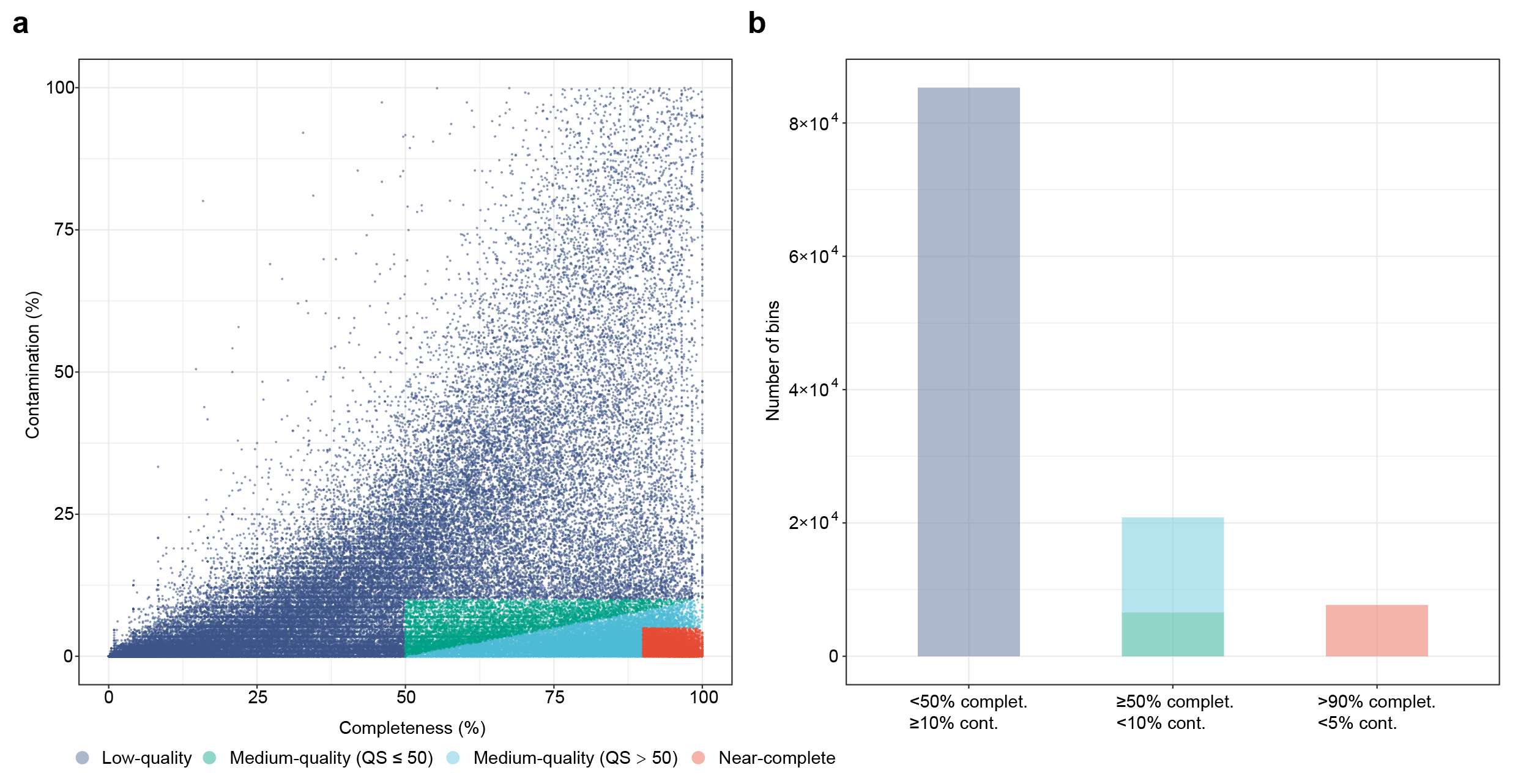


**Fig. S9 CheckM quality assessment. a** Quality assessment of the 116,138 MAGs. **b** Number of bins recovered according to the level of genome completeness and contamination. low-quality, <50% completeness or ≥10% contamination; medium-quality, ≥50% completeness and <10% contamination; near-complete, >90% completeness and <5% contamination. Quality score (QS) = completeness − 5 × contamination.


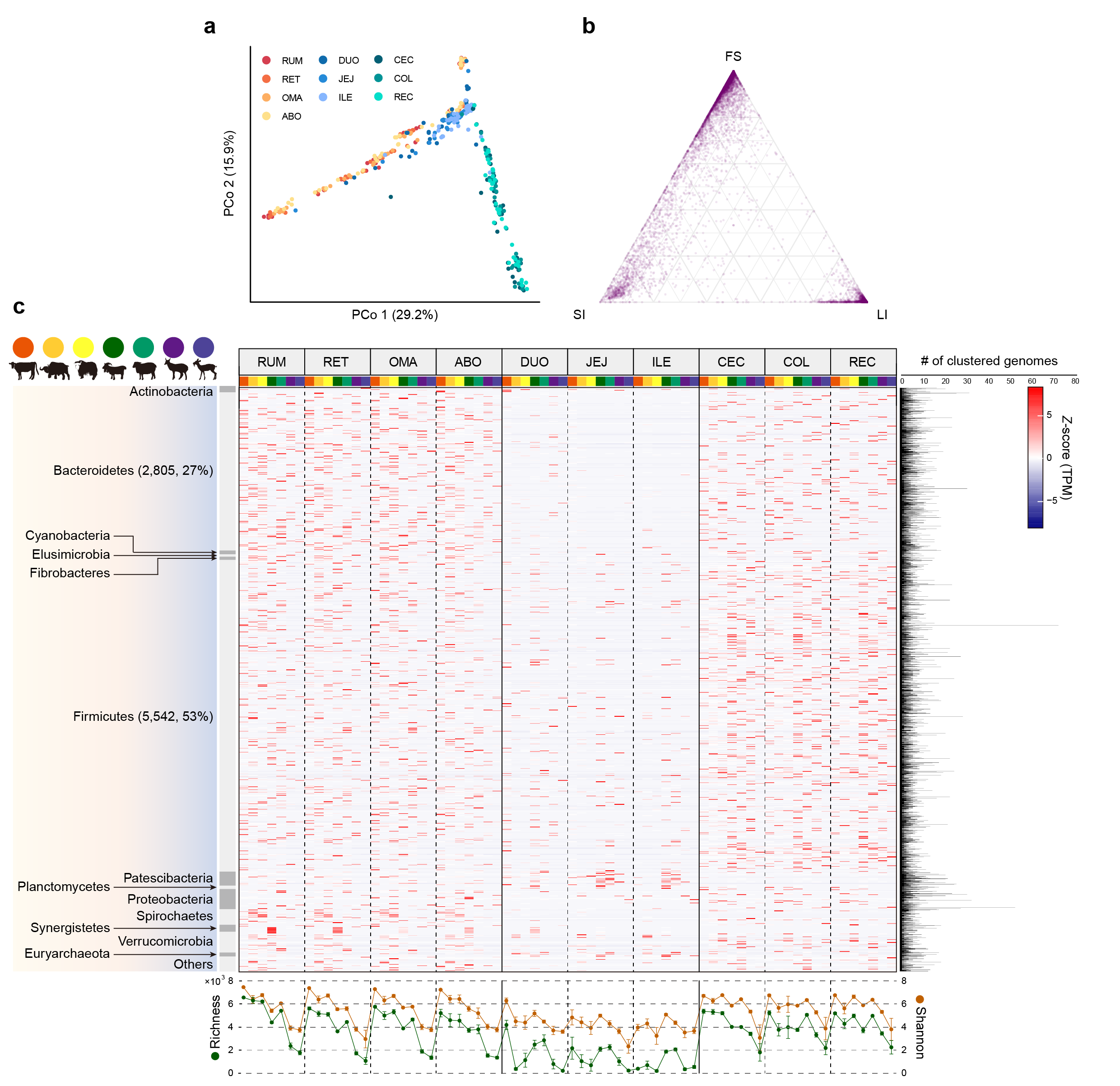


**Fig. S10 Distribution of 10,373 genomes across the ruminant GIT.** **a** PCoA plot based on differences in the abundances of MAGs across GIT regions according to the Bray-Curtis dissimilarity index. RUM, rumen; RET, reticulum; OMA, omasum; ABO, abomasum; DUO, duodenum; JEJ, jejunum; ILE, ileum; CEC, cecum; COL, colon; REC, rectum. **b** Ternary plots showing differences in abundances of MAGs between the stomach (FS), small intestine (SI), and large intestine (LI). **c** Heat map showing variations in the abundances of the MAGs (with dominant phyla assigned to the MAGs to the left and numbers of clustered genomes in each MAG, according to dereplication with ≥99% average nucleotide identity to the right), with alpha diversity (richness and Shannon index) shown in the lower graph.


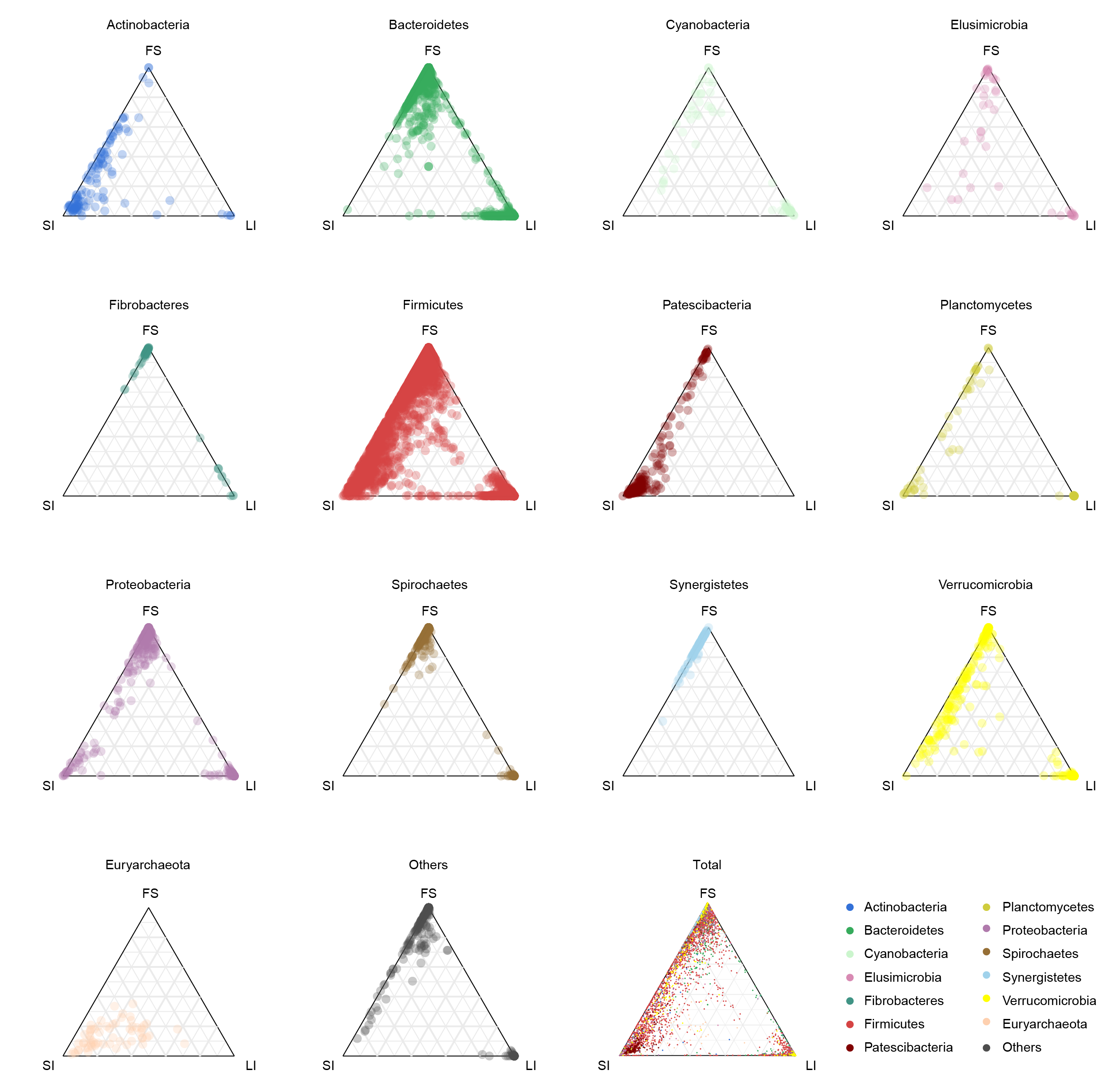


**Fig. S11 Variations in enrichment of MAGs among GIT regions.** Ternary plots showing differences in the relative abundances of the MAGs assigned to the indicated phyla in the stomach (FS), small intestine (SI), and large intestine (LI). The circle color indicates the phylum classification, and the circle size indicates the average abundance of MAGs among GIT regions.


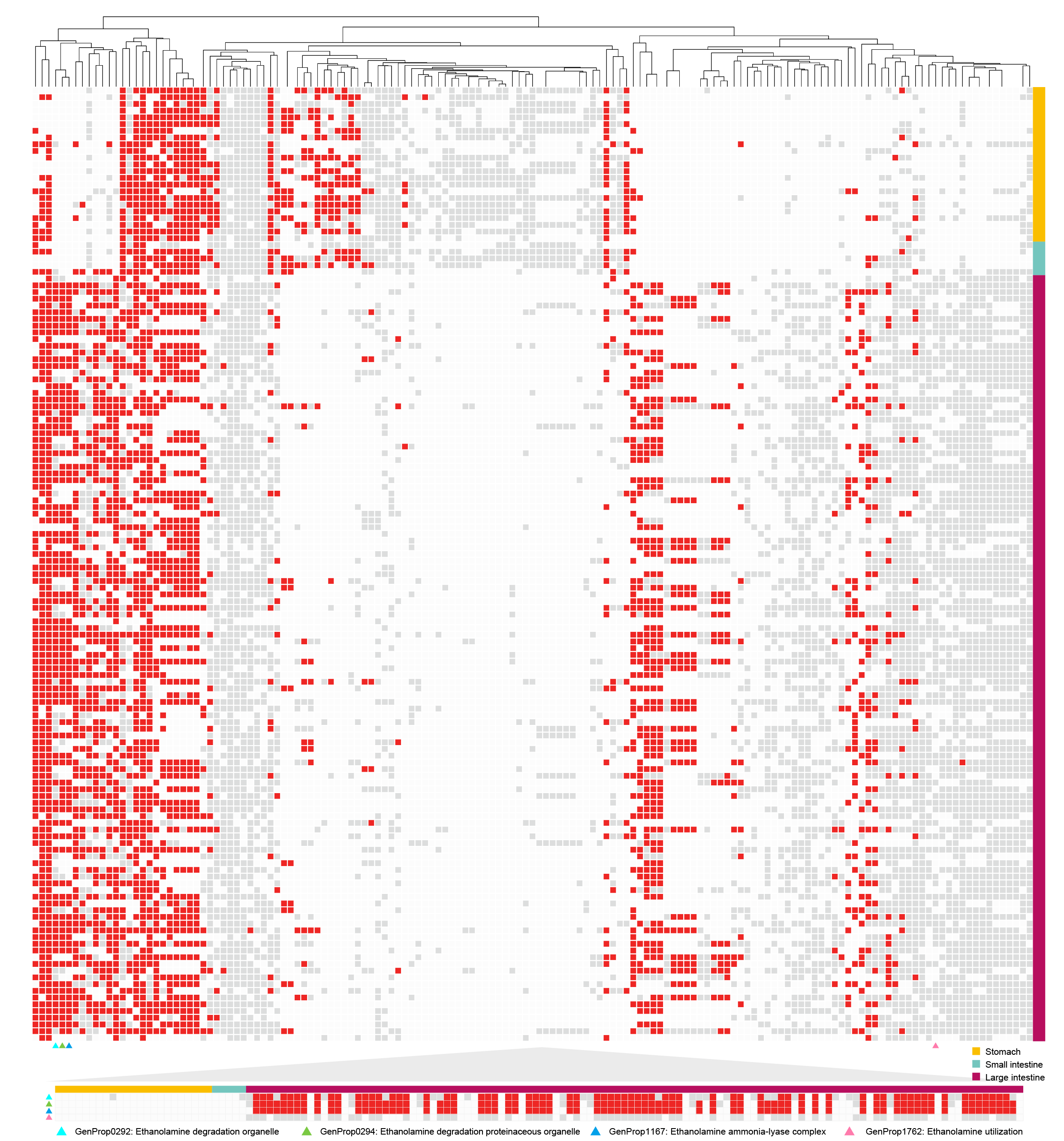


**Fig. S12 Comparative analysis of genomes of the CAG-110 genus.** Results of an analysis based on the GPs assigned to each of the 142 genomes detected in the sampled GIT regions (stomach, small intestine, and large intestine; *n* = 23, 5 and 114, respectively) assigned to the genus CAG-110. GPs classified as complete and partial are colored red and grey, respectively. The bottom shows the four GPs related to ethanolamine degradation and utilization, including GenProp0292 (ethanolamine degradation organelle), GenProp0294 (ethanolamine degradation proteinaceous organelle), GenProp1167 (ethanolamine ammonia-lyase complex) and GenProp1762 (ethanolamine utilization).


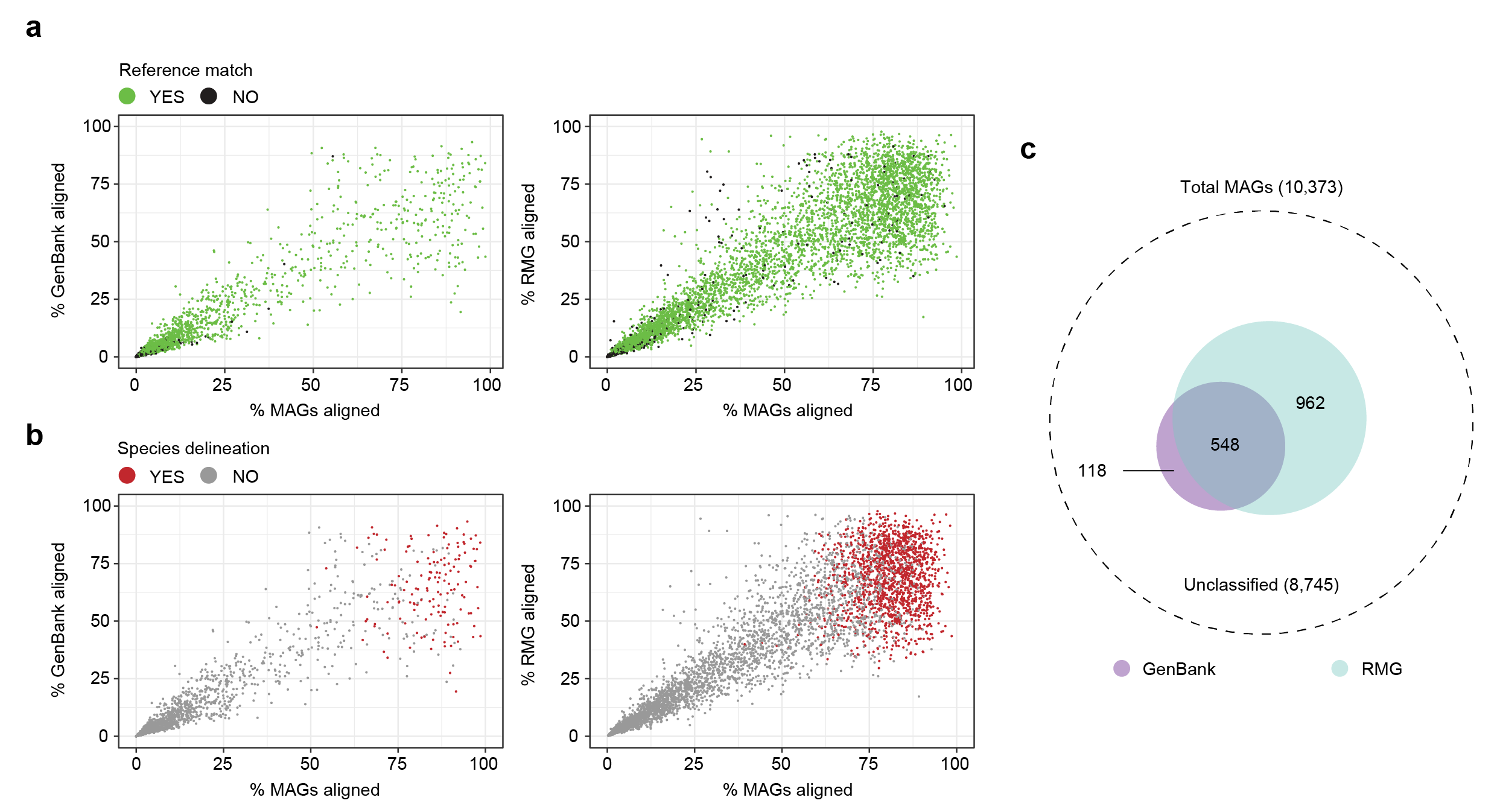


**Fig. S13 Species-level clustering of reference genomes and MAGs. a** Percentages of the 10,373 MAGs that did and did not match entries in the GenBank database and the published RMG dataset (colored green and black, respectively) at the indicated alignment levels. **b** MAGs that were classified at the species level in the GenBank database and the published RMG dataset based on the threshold of ≥95% average nucleotide identity and ≥60% fraction alignment. Red and grey points indicate that the MAGs were above and under the species threshold, respectively. **c** Summary of the matching results: 8,745 USGs were obtained.


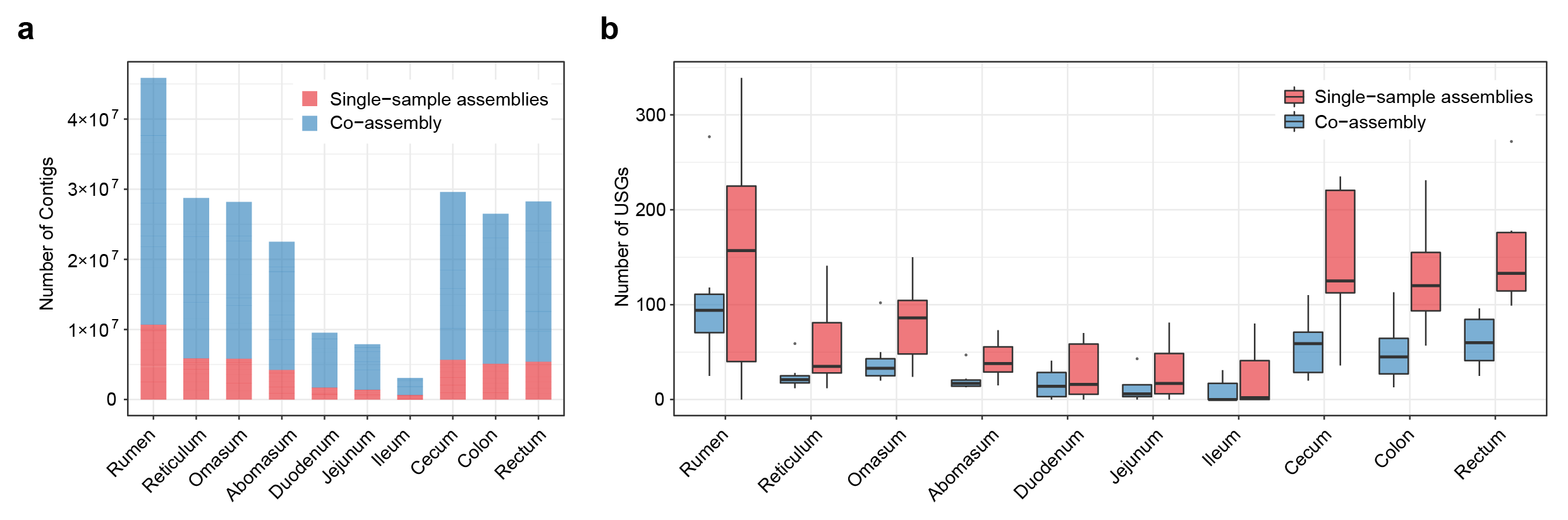


**Fig. S14 Distribution of the 8,745 USGs across the ruminant GIT.** **a** Numbers of contigs originating from single-sample assemblies and co-assembly across the GIT regions. **b** Numbers of USGs originating from single-sample binning and co-assembly binning across the GIT regions.


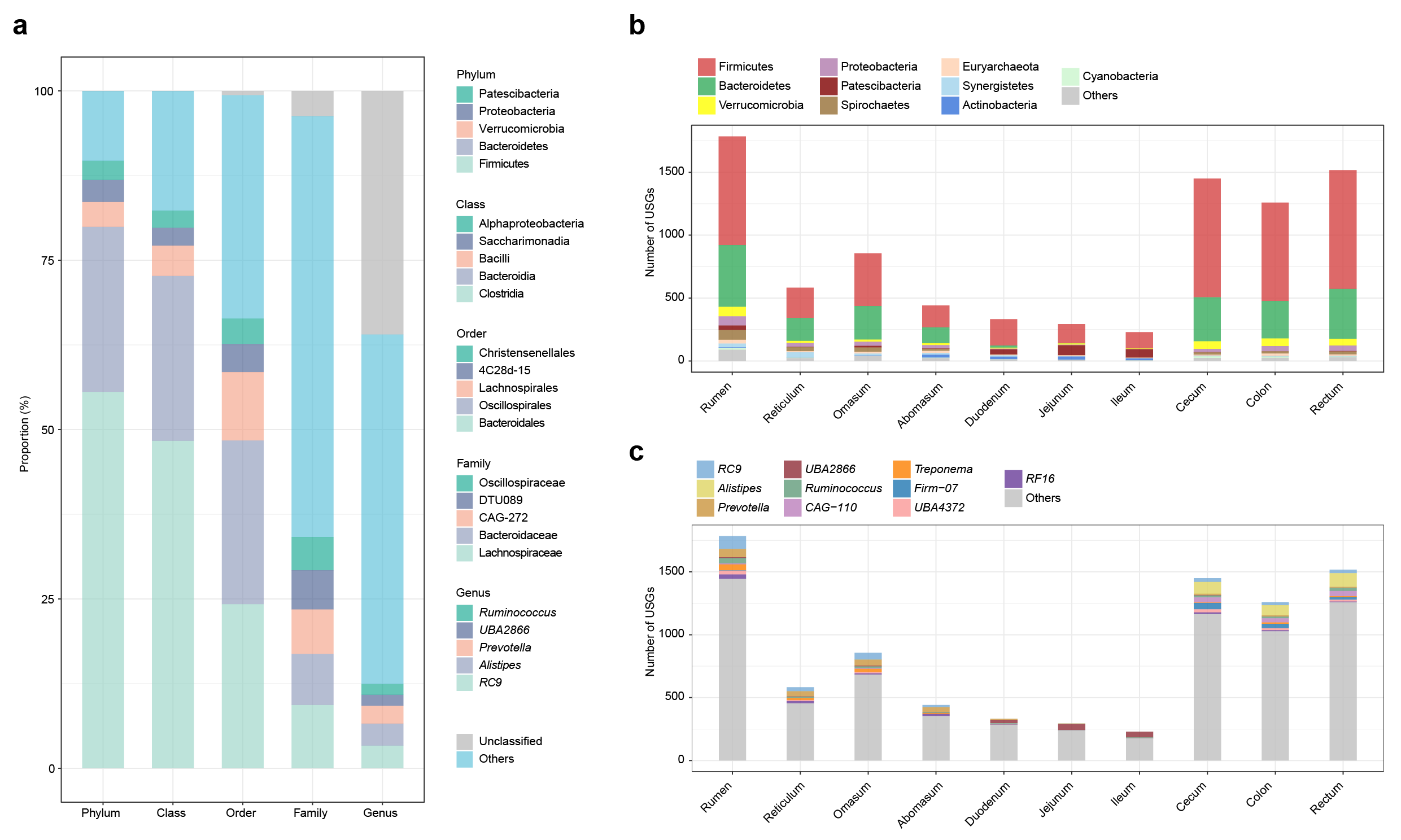


**Fig. S15 Taxonomic composition of the 8,745 USGs.** **a** Frequencies of phyla, classes, orders, families, and genera among the USGs (increasing from top to bottom). The five most frequently observed taxa of each rank are shown in the legend, with the remainder grouped as ‘others’ and ‘unclassified’. Taxonomic composition of the USGs across the GIT regions at the phylum **b** and genus **c** levels. The 10 most frequently observed taxa are shown in the legend, with the remaining grouped as ‘others’.


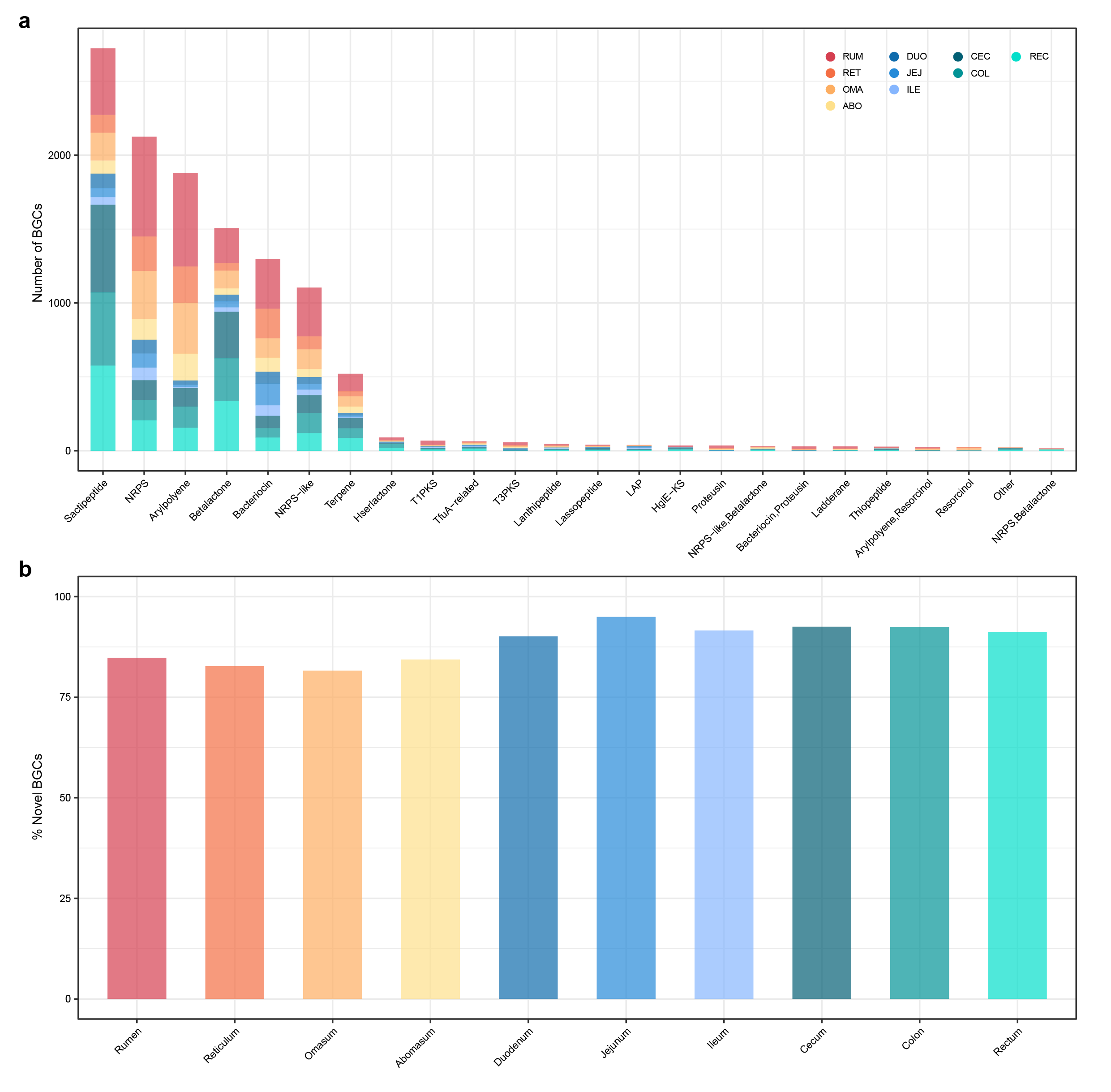


**Fig. S16 Biosynthetic gene clusters found in the human gut species. a** Number of secondary metabolite biosynthetic gene clusters (BGCs) found in the USGs from GIT regions, subdivided by functional category. Only the 24 most abundant categories are depicted. NRPS, nonribosomal peptide synthetase; PKS, polyketide synthases; LAP, linear azol(in)e-containing peptides. **b** Fraction of BGCs detected in the USGs from GIT regions that did not match the Minimum Information about a Biosynthetic Gene cluster (MIBiG) database.


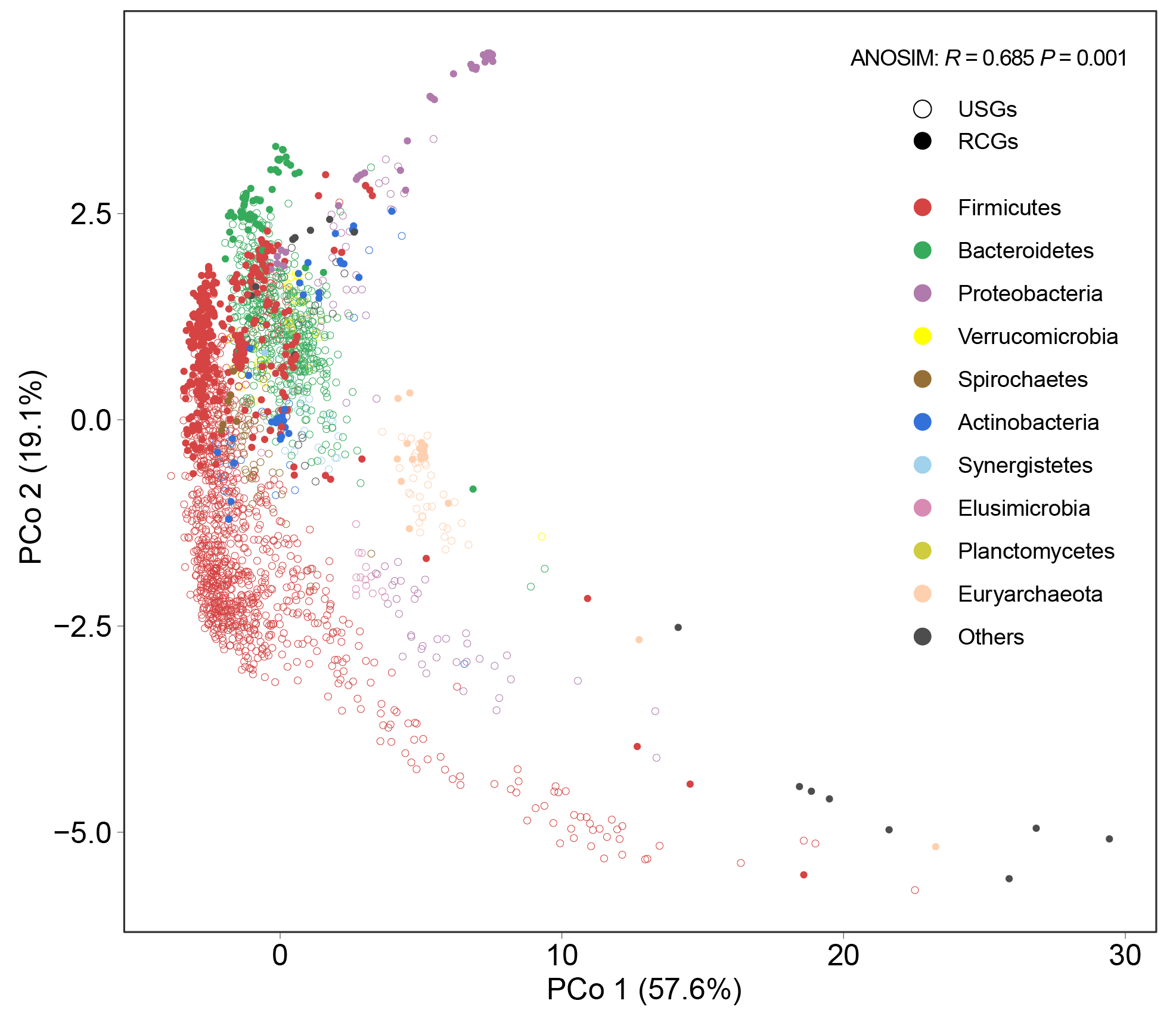


**Fig. S17 Differences in GP profiles between the USGs and RCGs.** PCoA plot of the GP profiles generated based on the Bray-Curtis dissimilarities, with the color of circles indicating the assigned phyla of these genomes. The 10 most frequent phyla are shown in the legend, with the remaining grouped as ‘others’. The difference between the USGs and RCGs was assessed by ANOSIM. RCGs, a collection of ruminant cultured genomes.


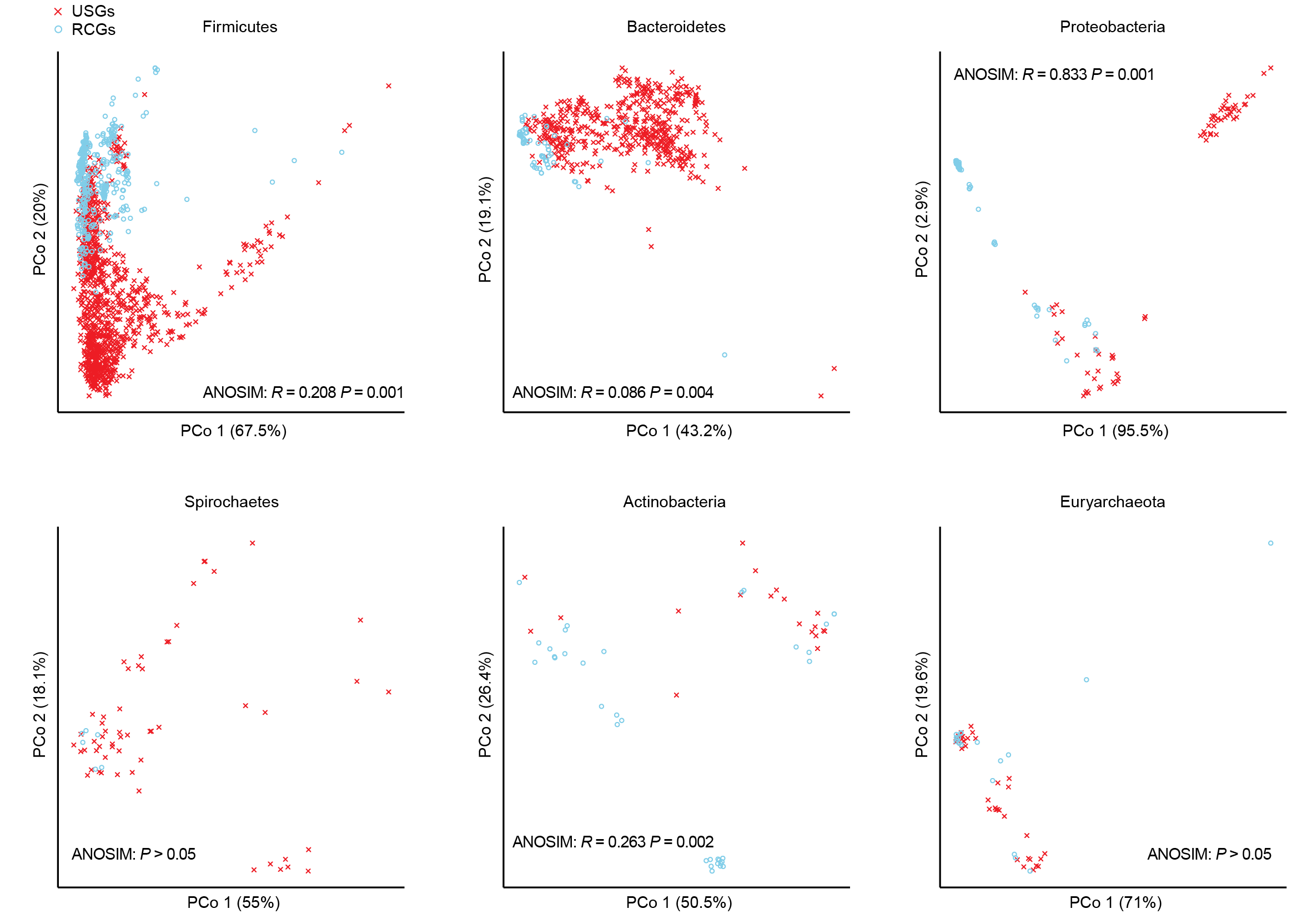


**Fig. S18 Comparison of the USGs and RCGs in the prevalent phyla.** PCoA plots based on the Bray-Curtis dissimilarities of GPs between the USGs and RCGs assigned to the five most prevalent phyla (Firmicutes, Bacteroidetes, Proteobacteria, Spirochaetes, Actinobacteria and Euryarchaeota). The difference between the USGs and RCGs were assessed by ANOSIM.


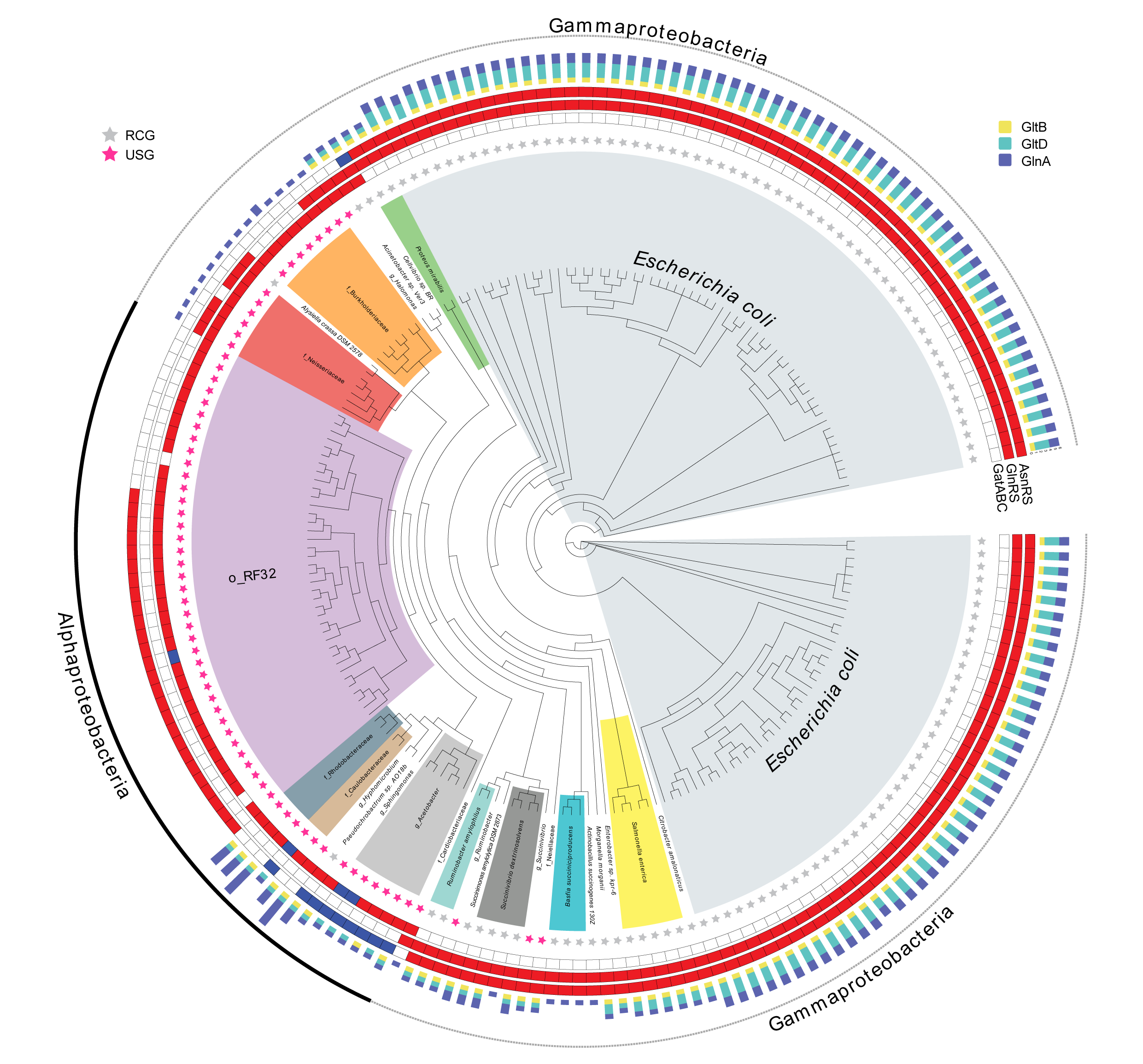


**Fig. S19 Phylogenetic tree of the 194 proteobacteria genomes.** The tree is labelled according to the taxa assigned to the Alphaproteobacteria and Gammaproteobacteria classes. Colored stars represent clades from USGs or RCGs. Complete and partial GPs related to protein synthesis are colored red and blue, respectively, including GlnRS (GenProp0189, tRNA-Gln direct aminoacylation), GsnRS (GenProp0259, tRNA-Asn direct aminoacylation) and GatABC (GenProp0188, GatABC aspartyl/glutamyl-tRNA amidotransferase complex). Bar graphs in the outer layer show numbers of KO genes related to glutamate synthesis matching those of the corresponding genomes. GltB, glutamate synthase [NADPH] large chain; GltD, glutamate synthase [NADPH] small chain; GlnA, glutamine synthetase.


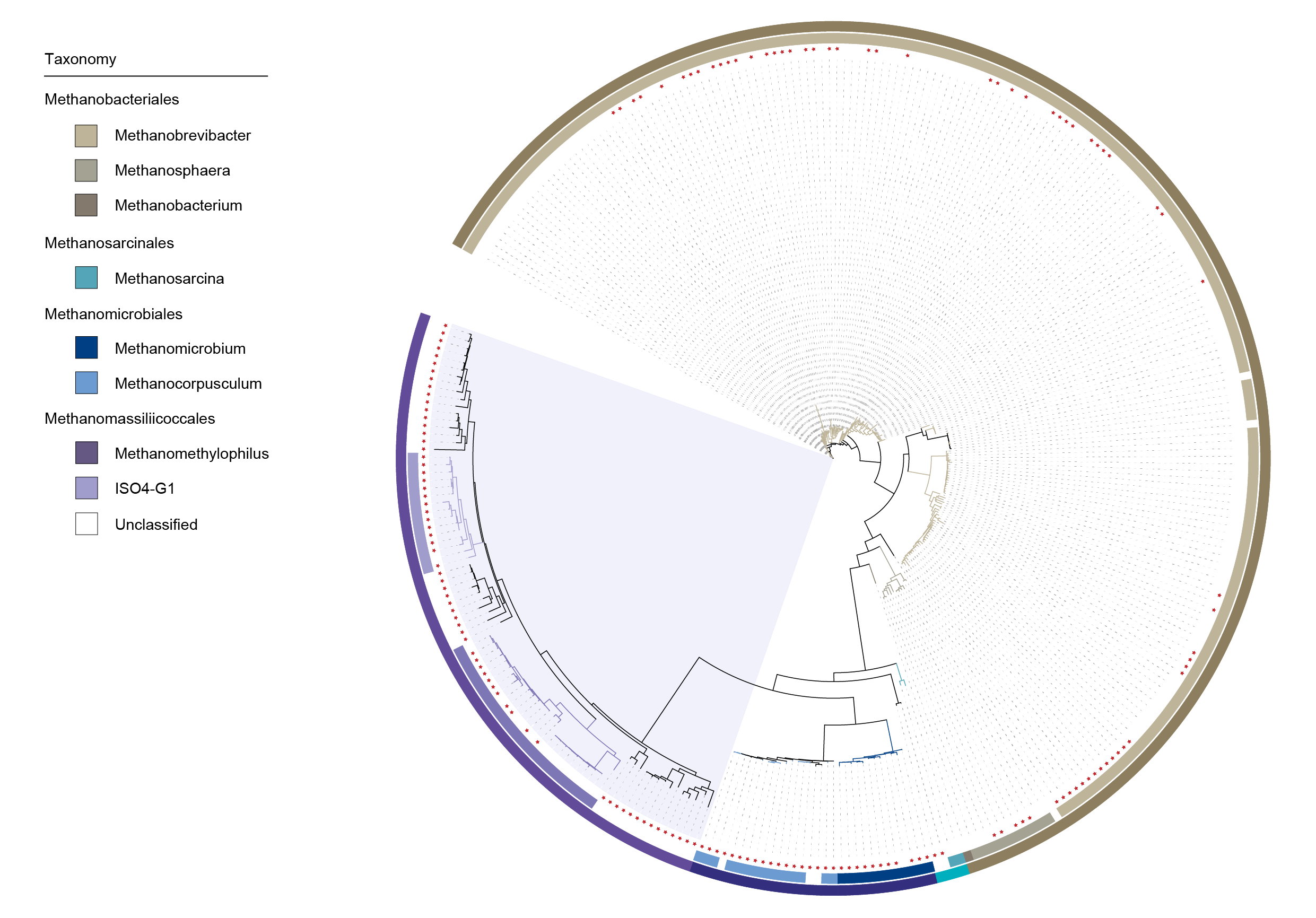


**Fig. S20 Phylogenetic tree of mutualistic archaea.** The maximum-likelihood tree of the 318 archaeal genomes, including 160 MAGs from this study and 158 previously published genomes, was constructed using PhyloPhlAn [4] and represents a phylogeny of GIT mutualistic archaea. Stars indicate the MAGs reconstructed in this study. Clades are labelled according to genus and order.


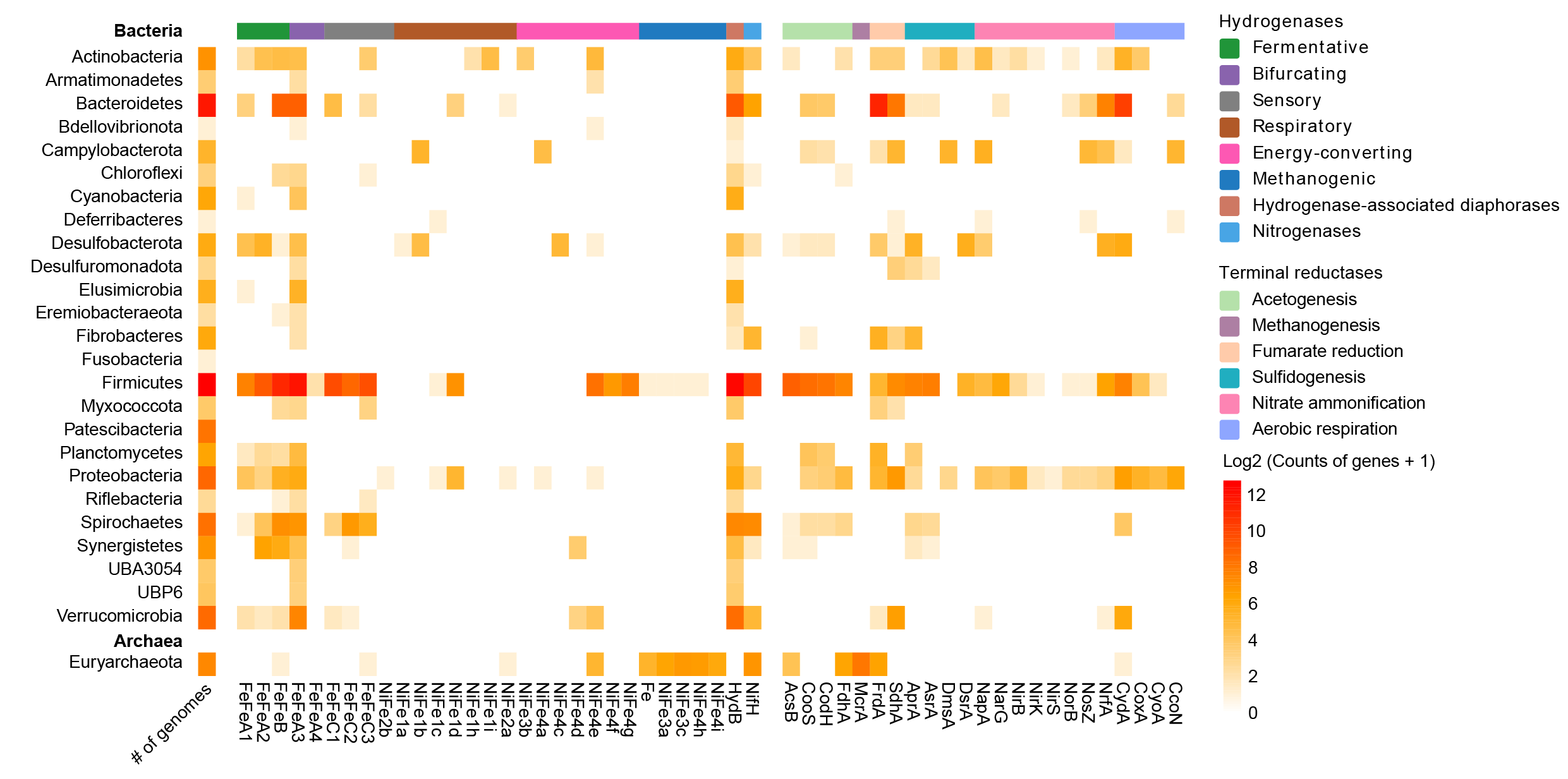


**Fig. S21 Distributions of hydrogenases and associated terminal reductases in the 10,373 MAGs.** Distributions (in phyla with hydrogenase-encoding genes) of fermentative hydrogenases (group A1, A2 and B FeFe-hydrogenases), bifurcating hydrogenases (group A3 and A4 FeFe-hydrogenases), sensory hydrogenases (group C FeFe-hydrogenases and group 2b NiFe-hydrogenases), respiratory hydrogenases (group 1a, 1b, 1c, 1d, 1h, 1i and 2a NiFe-hydrogenases), energy-converting hydrogenases (bidirectional; group 3b, 4a, 4c, 4d, 4e, 4f and 4g NiFe-hydrogenases) and methanogenic hydrogenases (Fe-hydrogenases, group 3a, 3c, 4h and 4i NiFe-hydrogenases). *HydB*, hydrogenase-associated diaphorase. *NifH*, nitrogenase. The H_2_ uptake pathway includes genes involved in acetogenesis (*AcsB*, acetyl-CoA synthase; *CooS* and *CodH*, anaerobic carbon-monoxide dehydrogenase; *FdhA*, formate oxidation), methanogenesis (*McrA*, methyl-CoM reductase), fumarate reduction (*FrdA* and *SdhA*, fumarate reductase), sulfidogenesis (*AprA*, adenylylsulfate reductase; *AsrA*, alternative sulfite reductase; *DmsA*, DMSO and TMAO reductase, *DsrA*, dissimilatory sulfite reductase), nitrate ammonification (*NapA*, periplasmic nitrate reductase; *NarG*, dissimilatory nitrate reductase; *NirB*, *NirK* and *NirS*, nitrite reductase; *NorB*, nitric oxide reductase; *NosZ*, nitrous oxide reductase; *NrfA*, ammonia-forming nitrite reductase), and aerobic respiration (*CydA*, cytochrome bd oxidase; *CoxA*, *CyoA* and *CcoN*, cytochrome c oxidase).


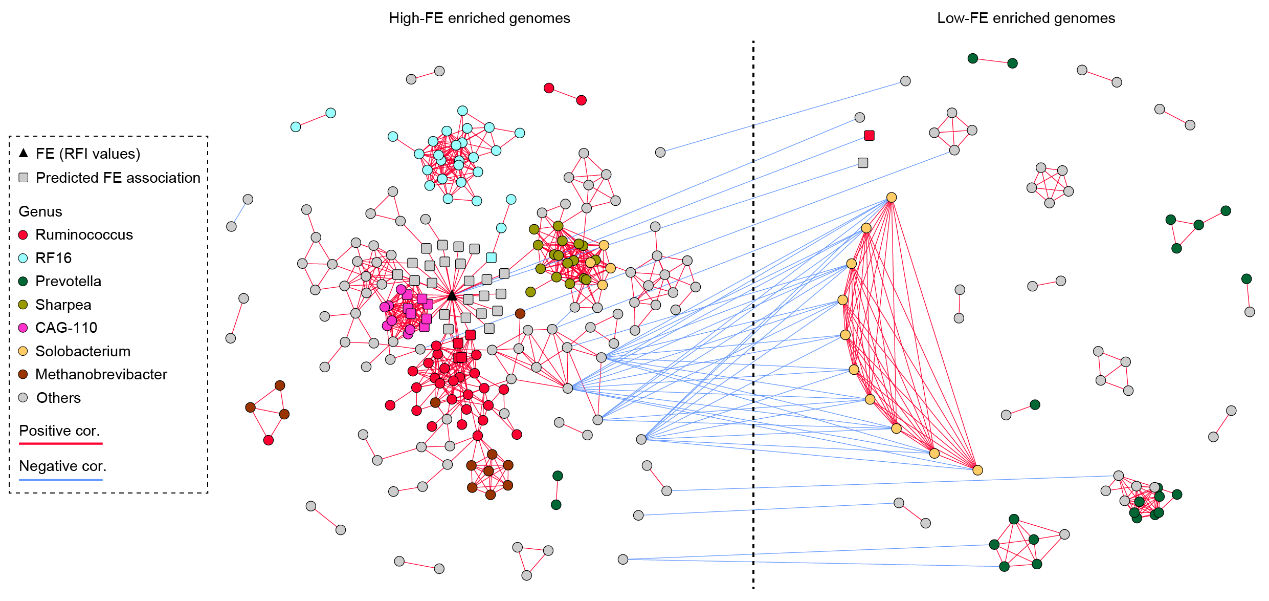
**Fig. S22 Associations of GIT microbial species with cattle feed efficiency (FE).** Correlation network between FE and the 410 differentially enriched genomes identified by differential analysis (log_2_ ^fold-change^ > 1 and *P* < 0.05). Nodes to the left and right of the dotted line indicate genomes enriched in high- and low-FE samples, respectively. Red and blue lines indicate nodes with Pearson correlation coefficients >0.5 and <−0.5, respectively. Circles are colored according to the annotated genera and prevalent genera are shown in the legend, with the remaining grouped as ‘others’. The squares show the 37 detected genomes associated with FE traits.

**References**

1. Hess M, Sczyrba A, Egan R, Kim TW, Chokhawala H, Schroth G, et al. Metagenomic discovery of biomass-degrading genes and genomes from cow rumen. Science. 2011;331: 463-467.

2. Li J, Zhong H, Ramayo-Caldas Y, Terrapon N, Lombard V, Potocki-Veronese G, et al. A catalog of microbial genes from the bovine rumen unveils a specialized and diverse biomass-degrading environment. Gigascience. 2020;9:giaa057.

3. Stewart RD, Auffret MD, Warr A, Walker AW, Roehe R, Watson M. Compendium of 4,941 rumen metagenome-assembled genomes for rumen microbiome biology and enzyme discovery. Nat Biotechnol. 2019;37:953-961.

4. Segata N, Börnigen D, Morgan XC, Huttenhower C. PhyloPhlAn is a new method for improved phylogenetic and taxonomic placement of microbes. Nat Commun. 2013;4:2304.
